# Comprehensive Comparative Genomics and Phenotyping of *Methylobacterium* Species

**DOI:** 10.3389/fmicb.2021.740610

**Published:** 2021-10-06

**Authors:** Ola Alessa, Yoshitoshi Ogura, Yoshiko Fujitani, Hideto Takami, Tetsuya Hayashi, Nurettin Sahin, Akio Tani

**Affiliations:** ^1^Institute of Plant Science and Resources, Okayama University, Okayama, Japan; ^2^Division of Microbiology, Department of Infectious Medicine, Kurume University School of Medicine, Kurume, Japan; ^3^Atmosphere and Ocean Research Institute, The University of Tokyo, Kashiwa, Japan; ^4^Department of Bacteriology, Graduate School of Medical Sciences, Kyushu University, Fukuoka, Japan; ^5^Egitim Fakultesi, Mugla Sitki Kocman University, Mugla, Turkey

**Keywords:** *Methylobacterium*, comparative genomics, methylotroph, methanol dehydrogenase, *Methylorubrum*

## Abstract

The pink-pigmented facultative methylotrophs (PPFMs), a major bacterial group found in the plant phyllosphere, comprise two genera: *Methylobacterium* and *Methylorubrum.* They have been separated into three major clades: A, B (*Methylorubrum*), and C. Within these genera, however, some species lack either pigmentation or methylotrophy, which raises the question of what actually defines the PPFMs. The present study employed a comprehensive comparative genomics approach to reveal the phylogenetic relationship among the PPFMs and to explain the genotypic differences that confer their different phenotypes. We newly sequenced the genomes of 29 relevant-type strains to complete a dataset for almost all validly published species in the genera. Through comparative analysis, we revealed that methylotrophy, nitrate utilization, and anoxygenic photosynthesis are hallmarks differentiating the PPFMs from the other *Methylobacteriaceae*. The *Methylobacterium* species in clade A, including the type species *Methylobacterium organophilum*, were phylogenetically classified into six subclades, each possessing relatively high genomic homology and shared phenotypic characteristics. One of these subclades is phylogenetically close to *Methylorubrum* species; this finding led us to reunite the two genera into a single genus *Methylobacterium*. Clade C, meanwhile, is composed of phylogenetically distinct species that share relatively higher percent G+C content and larger genome sizes, including larger numbers of secondary metabolite clusters. Most species of clade C and some of clade A have the glutathione-dependent pathway for formaldehyde oxidation in addition to the H_4_MPT pathway. Some species cannot utilize methanol due to their lack of MxaF-type methanol dehydrogenase (MDH), but most harbor an XoxF-type MDH that enables growth on methanol in the presence of lanthanum. The genomes of PPFMs encode between two and seven (average 3.7) genes for pyrroloquinoline quinone-dependent alcohol dehydrogenases, and their phylogeny is distinctly correlated with their genomic phylogeny. All PPFMs were capable of synthesizing auxin and did not induce any immune response in rice cells. Other phenotypes including sugar utilization, antibiotic resistance, and antifungal activity correlated with their phylogenetic relationship. This study provides the first inclusive genotypic insight into the phylogeny and phenotypes of PPFMs.

## Introduction

Within the *Methylobacteriaceae* family lies a group of ubiquitous Gram-stain-negative bacteria known as the pink-pigmented facultative methylotrophs (PPFMs) due to their pink color and their ability to use reduced one-carbon (C1) compounds such as methanol, methylamine, formaldehyde, and formate as their sole energy and carbon source. Many PPFMs either live freely in water, soil, or air (Gallego et al., 2005; Park et al., 2018; Kim et al., 2020) or are associated with the rhizosphere (Grossi et al., 2020) or phyllosphere (Madhaiyan et al., 2011; Wellner et al., 2013); they represent a dominant bacterial group in the phyllosphere (Knief et al., 2008; Delmotte et al., 2009).

Pink-pigmented facultative methylotrophs colonize plants as epiphytes or endophytes (Verginer et al., 2010; Jorge et al., 2019), although in some cases they reside in the intracellular spaces of plant meristematic cells (Pirttilä et al., 2000) or root nodules (Jourand et al., 2004), where they establish a symbiotic relationship with the plant. They use methanol emitted from the plant, processing it by means of two paralogous methanol dehydrogenases (MDHs): MxaFI, a Ca^2+^-dependent MDH (Anthony et al., 1994; Blake et al., 1994), and XoxF, a lanthanide (Ln^3+^)-dependent MDH (Hibi et al., 2011; Nakagawa et al., 2012).

Methylotrophy is reported to be vital for PPFMs to inhabit and flourish in the phyllosphere (Sy et al., 2005). Methanol serves as a carbon and energy source for methylotrophic bacteria; in return, the bacteria produce phytohormones (auxin and cytokinin) and secondary metabolites (Ivanova et al., 2001; Lidstrom and Chistoserdova, 2002) and fix nitrogen (Jourand et al., 2004), contributing to plant growth (Abanda-Nkpwatt et al., 2006). They also generate fruit flavor (Verginer et al., 2010) and support plant tolerance of biotic and abiotic stresses (Ardanov et al., 2012; Jorge et al., 2019). *Methylobacterium* species are also found as symbionts of microalgae and mosses (Tani et al., 2012; Krug et al., 2020).

Taxonomically, the first PPFM strain described in the literature was isolated from earthworm contents; at that time, it was named “*Bacillus extorquens*” (Bassalik, 1913). In 1976, the genus *Methylobacterium* was established with *Methylobacterium organophilum* as the only species within it (Patt et al., 1976); since then, the number of known *Methylobacterium* species has risen to 63 (Hedlund et al., 2021; Maeng et al., 2021)^1^. Various attempts have been made to examine PPFMs from a taxonomic perspective (Green and Bousfield, 1982; Hood et al., 1987; Tsuji et al., 1990; Tani et al., 2015; Kröber et al., 2021b). Green and Ardley (2018) recently divided the *Methylobacterium* species into three clades (A, B, and C) based on phenotypic characteristics, multilocus sequence analysis (MLSA), and 16S rRNA gene sequence analysis. Clade A represents the core *Methylobacterium* species. A new genus *Methylorubrum* was proposed for the species in clade B, which includes the best-studied model strain *Methylorubrum extorquens* AM1. The species in clade C are considered species *incertae sedis*, as most of the species within it cannot utilize methanol, and some species have other unique abilities such as nodulation in legumes. A recent large study on entire alpha-proteobacterial genomes, however, suggested reclassifying the *Methylorubrum* species into *Methylobacterium* until additional genome data become available for PPFMs (Hördt et al., 2020).

Identification, classification, and species delineation of prokaryotes require various analyses of phenotypic features including morphological and biochemical characteristics, genomic DNA homology (DNA–DNA hybridization, DDH), genome GC% content (Schildkraut et al., 1961; McCarthy and Bolton, 1963; Johnson, 1973), and 16S rRNA gene polymorphism (Weisburg et al., 1991). Although the methodologies for these analyses are well established, the experimental outcomes may be unstable in practice. For example, DDH requires special experimental setups and depends highly on the quality and purity of genomic DNA. Fatty acid methyl ester (FAME) analysis is dramatically affected by culture conditions. 16S rRNA gene sequencing is widely used as a gold standard, but sequencing of the PCR product masks the intragenomic heterogeneity of the gene copies. On the other hand, the recent availability of genome data has paved the way for more robust high-throughput analyses of bacterial taxonomy. Sophisticated methods and tools including average nucleotide identity (ANI) and *in silico* (digital) DNA–DNA hybridization (dDDH), which have species cutoff values of 95–96% and 70%, respectively, are now replacing the old methods (Auch et al., 2010; Meier-Kolthoff et al., 2013; Jain et al., 2018; Meier-Kolthoff and Göker, 2019). Many annotation tools and pathway/module analysis pipelines for bacterial genomes have also been developed to characterize the genotypes of bacterial species. Nowadays, whole-genome shotgun sequencing has become more affordable, enabling us to compare large sets of genome data, which has shed light on the functions and evolutionary histories of various species through core- and pan-genome analysis (Tettelin et al., 2005; Chaudhari et al., 2016).

To date, no comprehensive comparative genomic analysis of *Methylobacterium* and *Methylorubrum* species has been performed due to the unavailability of genome assemblies of all validly published species. In this study, we sequenced the genomes of previously unsequenced and low-quality-type strains. Sixty-two genome assemblies representing almost all type strains were used for comparative genomic analyses. The genome data set was used to assess phylogeny based on each strain’s whole genome rather than any single gene. Using genome mining, we also tried to highlight important and experimentally verified phenotypic characteristics related to methylotrophy and plant symbiosis. This is the first report that covered all available PPFM genome information and analyzed the genome content alongside phenotyping data. Our work has yielded a more comprehensive understanding of the physiology and diversity of PPFMs.

## Materials and Methods

### DNA Extraction, Genome Sequencing, Annotation, and 16S rRNA Gene Phylogeny

All publicly available genome assemblies of type strains of *Methylobacterium* and *Methylorubrum* species (hereafter abbreviated as *Mb*. and *Mr.*, respectively) were downloaded from databases (NCBI and JGI) at the end of April 2021 (Supplementary Table 1). In this study, we newly sequenced the genomes of 23 type strains and six strains that have available assemblies but with low quality [*Methylobacterium dankookense* SW08-7^*T*^ (GCA_902141855.1), *Methylobacterium frigidaeris* GIER25-16^*T*^ (GCA_002759055.1), *Methylobacterium mesophilicum* SR1.6/6^*T*^ (GCA_000364445.2), *Methylobacterium crusticola* MIMD6^*T*^ (GCA_003574465.1), *Methylobacterium soli* YIM 48816^*T*^ (GCA_008806385.1), and *Methylorubrum thiocyanatum* JCM10893^*T*^ (GCA_001310875.1)]. The quality of the available assembly of *Mb. organophilum*^*T*^ (GCA_003096615.1) was sufficiently high, but the source strain DSM 760^*T*^ is believed to have been contaminated such that the available assembly did not accurately represent its taxonomic status (Kato et al., 2005; Volpiano et al., 2021). Therefore, the strain *Mb. organophilum* NBRC 15689^*T*^ was newly sequenced in this study.

The JGI bacterial DNA extraction CTAB protocol (William et al., 2004) was used to extract genomic DNA from cells grown on Reasoner’s 2A (R2A, Becton Dickinson) agar plates for 1 week. The genome sequencing was performed using Illumina MiSeq or MGI DNBseq. The assembling was performed using CLC Genomics Workbench 20.0.4 or SPAdes (Bankevich et al., 2012; Seemann, 2017) at Galaxy^2^ (Cuccuru et al., 2014; Afgan et al., 2018; Jalili et al., 2020). All assemblies were re-annotated using Prokka (Seemann, 2014) at Galaxy. The contigs encoding 16S rRNA genes were extracted using the ContEst16S tool (Lee et al., 2017), and their identity was checked at EzBioCloud database^3^. Contamination and completeness of the assemblies were checked using CheckM (v1.0.18, Parks et al., 2015) with thresholds of >90% for completeness and <5% for contamination. Sixty-two assemblies were used for further analysis. Additionally, 16 assemblies of other species within the family *Methylobacteriaceae* (15 genomes from the genus *Microvirga* and one from the genus *Enterovirga*) were added to the analysis as an out-group genome set.

The 16S rRNA-based phylogeny was created using the TYGS (Type Strain Genome Server) platform (Meier-Kolthoff and Göker, 2019)^4^; the analysis was restricted to the 62 PPFM assemblies and *Microvirga brassicacearum* (CDVBN77^*T*^, GCA_008757455.1) as an outgroup. iTOL was used for visualization of the tree (Letunic and Bork, 2021).

### Whole-Genome-Based Phylogeny Analysis and Pan-Genome Analysis

*In silico* DDH values were calculated in the TYGS platform using formula d_4_, which is the sum of all identities found in the high score segment pairs (HSPs) divided by the total length of all HSPs. The ANI was calculated using FastANI (Jain et al., 2018) at Galaxy. Pan-genome analysis of the PPFMs was performed using the Bacteria Pan Genome Analysis pipeline (BPGA, Chaudhari et al., 2016) with the amino acid sequences and default parameter settings (50% identity). The tree files provided by the BPGA pipeline and TYGS were visualized with iTOL.

### Functional Annotation

Kyoto encyclopedia of genes and genomes (KEGG) and COG functional clustering analysis was performed within the BPGA program to find the distribution of core and unique genes in the PPFMs. For pathway/module analysis, the metabolic and physiological potential evaluator Genomaple^TM^ pipeline was used (version 2.3.2, Takami et al., 2016; Arai et al., 2018). The analysis included the genomes of 78 species, namely, 16 outgroup species from the family *Methylobacteriaceae* and the 62 PPFM species. The module completion ratio (MCR) within the whole community (WC, regarded as an individual genome in this study) was used to measure the completeness of a module. Only modules that occurred in at least four but fewer than 65 genomes were taken into consideration; 90 modules met this criterion. Data was converted to a presence-and-absence binary matrix and visualized with R heatmap.3 package (Ihaka and Gentleman, 1996). Gene clusters for secondary metabolite synthesis were mined using antiSMASH pipeline version 6 (Medema et al., 2011; Blin et al., 2019) under the default settings.

### Genomics of Methylotrophy, Phyllosphere Adaptation, and Plant Colonization

The genes responsible for utilization of methanol and methylamine were extracted from the reference genome of the model organism *Mr. extorquens* AM1 (GCA_000083545.1) (Vuilleumier et al., 2009). The genes encoding the N-methylglutamate (NMG) pathway for methylamine utilization were from *Mb. extorquens* PA1 (GCA_000018845.1). Dichloromethane utilization genes were from *Methylobacterium dichloromethanicum* subsp. *dichloromethanicum* DM4 (GCA_000083545.1). The genes involved in adaptation to the phyllosphere and plant growth promotion [PGP, protection against UV damage and oxidative stress, biosynthesis of cytokinin, aminocyclopropane-1-carboxylic acid (ACC) deaminase, and chitinase] were from *Methylobacterium oryzae* CBMB20^*T*^ (GCA_000757795.1, Kwak et al., 2014) and *Methylobacterium aquaticum* MA-22A (GCA_001548015.1, Tani et al., 2015). Enzymes related to plant colonization (pectate lyase, cellulase, and endoglucanase) were from *Mr. extorquens* AM1 and *Mb. aquaticum* MA-22A. The genes for IAA production through the L-tryptophan pathway (monoamine oxidase, L-tryptophan decarboxylase, and aldehyde dehydrogenase) were from *Methylobacterium nodulans* ORS 2060^*T*^ (GCA_000022085.1).

The amino acid sequences of the genes of interest were used as queries for the BlastP analysis (50% identity threshold) against genome data of the strains. The resultant gene presence/absence table was used to examine the conservation of the genes. The duplicates were removed, and from each genome only the hit with the highest identity value was kept. Additional checking was done for *xoxF* to confirm that it existed in its complete cluster form, since each genome contained multiple copies of *xoxF* homologs. Reference sequence source accession numbers and annotation IDs are provided in Supplementary Table 2.

### Phylogenetic Tree Construction of Pyrroloquinoline Quinone-Dependent Alcohol Dehydrogenase Homologs

As mentioned above, one genome may contain multiple copies of pyrroloquinoline quinone dependent alcohol dehydrogenase (PQQ-ADH) homologs. To examine the ADH types encoded in each genome, the amino acid sequences of various ADHs (49 sequences, Supplementary Table 3) from different sources were manually picked up from the tree presented by Keltjens et al. (2014) and used as queries for a BlastP-search. Homologs with <50% identity and >0.001 *e*-value were removed. After duplicates had been removed, the Blast hit of the 252 sequences were aligned with MAFFT together with the reference sequences. A maximum likelihood phylogenetic tree was constructed using the FastTree (Price et al., 2010) at Galaxy, and tree annotation and visualization were performed with iTOL.

### Phenotypic Characteristics

For phenotypic analysis, of the 63 validly published *Methylobacterium* and *Methylorubrum* species type strains, we did not use *Methylorubrum populi* BJ001^*T*^ (a patented strain), *Methylorubrum pseudosasae* NBRC105205^*T*^ (contaminated culture), *Methylobacterium symbioticum* SB0023/3^*T*^, *Methylobacterium ajmalii* IF7SW-B2^*T*^, and *Methylobacterium radiodurans* 17Sr1-43^*T*^ (which were published recently). Additionally, we included *Mb. dichloromethanicum* subsp. *chloromethanicum* CM4, *Mb. dichloromethanicum* subsp. *dichloromethanicum* DM4, *Mr. extorquens* AM1 (ATCC 14718), and *Methylorubrum lusitanum* (NCIMB 13779 and DSM-14457) which is a later synonym of *Methylorubrum rhodesianum* DSM 5687^*T*^.

The strains were subjected to the following tests: growth on solidified nutrient broth (NB, Eiken Chemical), R2A agar (Difco^TM^), and Potato Dextrose agar (PDA, Difco^TM^); utilization of methanol, methylamine, dichloromethane, glycerol, ethanol, glucose, fructose, and sucrose, which was tested on a mineral medium (MM) solidified with 1.5% agar (Supplementary Table 4); salt tolerance (1% NaCl using glycerol as a carbon source); ampicillin resistance (25 and 50 mg/l on R2A); vitamin requirement (MM with methanol or glycerol as a carbon source without the vitamin mixture); and the effect of LaCl_3_ (30 μM) supplementation on methanol and glycerol growth. A 1-week-old colony of each type species grown on R2A agar was picked and streaked on an MM agar test plate. Plates were incubated at 28°C for 10 days. The carbon source concentrations were set to 0.5% (w/v for solid substances or w/w for liquid substances) except in the dichloromethane test, for which we inoculated the strains on solidified MM medium prepared in 12-well plates with a hole in each plate cover, and a small beaker containing 2 ml dichloromethane was placed in a 22.6-l glass desiccator. Cultivation took place in the dark. The growth was evaluated as either positive or negative after 10 days of incubation at 28°C. Oligotrophic growth on MM agar plates with no added carbon source was regarded as a negative control.

API 20NE strips were used according to the manufacturer’s instructions. The strips were incubated at 28°C and monitored for 7 days. Positive growth for the first three tests (reduction of nitrates, production of indole, and fermentation of glucose) was recorded after 24 h of inoculation. The remaining tests were checked on the second, third, fifth, and seventh days, but only the result on the final day was recorded.

### Antifungal Activity

Growth inhibition assay against *Fusarium oxysporum* f. sp. *lycopersici* strain (9859-1) race 2 was done as follows. PPFM strains that were capable of growing on PDA were tested. Fresh bacterial cells grown on R2A plates for 7 days were suspended in 0.9% NaCl at OD600 = 1.0, and 10 μl of the suspension was spread with a loop over half of a PDA solid medium surface. An agar plug (5 mm × 5 mm) containing *Fusarium* mycelium grown on PDA for 7 days was placed in the middle of each plate (technical triplicates). After 7–10 days at 25°C, plates were photographed and the fungal growth inhibition zone was estimated using ImageJ. The inhibition percentage was measured by comparing the fungal growth radius in the inoculated half of the plate (ir) with that in the non-inoculated half (cr) as follows:


Inhibition%=100×(cr-ir)/cr

### Indole Acetic Acid Production Through the L-Tryptophan Pathway

Bacterial cells grown on R2A plates were suspended in 0.9% NaCl (OD600 = 1.0), and 10 μl of the suspension was added to 1 ml of King’s B medium, which was composed of 20 g/l protease peptone, 1.15 g/l K_2_HPO_4_, and 1.5 g/l MgSO_4_ supplemented with 0.51 g/l L-tryptophan, 0.5% (w/v) glucose, and 0.5% (v/v) methanol prepared in 12-well cell culture plates. Cultivation was done at 28°C for 7 days with rotary shaking (300 rpm). One milliliter of the culture was centrifuged (9,100 × *g*) for 5 min at room temperature, and 100 μl of the supernatant was added to 100 μl of R1 reagent (12 g/l FeCl_3_ and 7.9 M H_2_SO_4_). The absorbance at 530 nm was measured after 30 min. Authentic IAA (1–100 μg/ml) was used as the standard. *Pseudomonas syringae* strain DC3000 (ATCC BAA-871) was used as a positive control. The experiment was performed in technical triplicate.

### Elicitation of Rice Cells

The PPFM strains were tested for defense elicitation in rice cells as described previously (Shinya et al., 2016). In short, 40 mg fresh rice cells grown in N6 liquid medium for 4 days at 25°C were suspended in 995 μl N6 medium in 24-well plates. Bacterial suspension (5 μl, OD600 = 1.0) was then added. *P. syringae* DC3000, chitin oligomer (GlcNAc)_8_ at a final concentration of 10 nM, and *Serratia marcescens* subsp. *marcescens* isolates 1–7 and 4–24, each of which has been shown to elicit a response previously (Wari et al., 2019), were used as positive controls. The plates were incubated at 25°C with shaking (300 rpm) and photographed at 24 and 40 h post-inoculation. The experiment was repeated two times. Defensive response was recorded as positive if the rice cells changed color, which indicates the accumulation of defense metabolites.

### Data Availability

The genome sequence data are deposited in the DNA data bank of Japan (DDBJ) under BioProject number PRJDB11873. The genome annotation information (GFF format) is provided as a Supplementary Material.

## Results

### General Considerations

In this study, we aimed to complete the genome information on this taxonomically heterogeneous group of methylotrophic bacteria and compare their genotypes and phenotypes. We collected strains from culture collections and genome assembly data from the databases. At the time we started the project, there were 63 *Methylobacterium* and *Methylorubrum* species validly published^5^. We initially intended to analyze all of these, but the sample of *Mr. pseudosasae* strain NBRC 105205^*T*^ we obtained was found to be contaminated, and its genome assembly was unavailable from public data repositories, so we excluded it from the analysis. For two of the remaining 62 species, we used cultures of non-type strains, namely, *Methylobacterium trifolii* strain TA88+4-73 (DSM 23632) and *Methylobacterium thuringiense* strain C61+2-34 (DSM 23674), both of which are closely related to the respective type strains *Mb. trifolii* strain TA73^*T*^ and *Mb. thuringiense* strain C34^*T*^. All other strains were type strains of the species. For phenotypic analysis, however, non-type strains were also used (details below). We PCR-amplified 16S rRNA gene fragments of all strains to confirm their identity before starting genome sequencing and phenotypic analyses.

### Genome Assembly, Annotation, and Quality Check

For 33 of the 62 *Methylobacterium* and *Methylorubrum* species type strains used in this study, we downloaded genome assemblies from GenBank (Supplementary Table 1). We sequenced the other 29 strains, 23 of which are reported for the first time in this study; the remaining six had publicly available assemblies, but these were of low quality. Information on the quality of each newly sequenced assembly is summarized in Supplementary Table 5. To ensure consistent protein coding sequences (CDS) analysis, all genome assemblies were re-annotated with Prokka, and quality-checked with CheckM. The genome size of each species ranged from 4.4 to 8.8 Mbases with CDSs ranging from 4,171 to 8,622 and GC% ranging from 65.9 to 72.7%. The average completeness was 98.8%, and the average contamination level was 0.4%. The 16S rRNA gene sequences extracted from the assemblies by ContEst16S were compared with the corresponding PCR-based sequences available in the EzBioCloud database. The 16S rRNA contig from *Methylobacterium terricola* 17Sr1-39^*T*^ was short (1,251 bp), but its identity was sufficiently high (99.5%). The 16S rRNA contig from *Methylobacterium planium* YIM 132548^*T*^ was also short (1,208 bp) with good identity (99.75%). One of the two 16S rRNA contigs from *Methylobacterium oryzihabitans* TER-1^*T*^ showed high homology to *Pseudomonas asplenii* ATCC 23835, suggesting an apparent contamination. Three assemblies, *Mb. ajmalii* IF7SW-B2^*T*^, *Methylobacterium segetis* 17J42-1^*T*^, and *Methylorubrum salsuginis* CGMCC 1.6474^*T*^, did not contain any 16S rRNA gene contig. We amplified and sequenced the PCR-amplified 16S rRNA gene fragments from the strains we obtained and confirmed their identity to those of the database. Overall, therefore, the genome assemblies were generally of good quality for comparative genomics, and we concluded that we could use the cultures for phenotypic analysis.

### 16S rRNA Gene-Based Phylogeny

The phylogenetic tree that we created in this study, based on 16S rRNA gene sequences extracted from the assemblies using the TYGS web tool, is shown in Supplementary Figure 1. This tree divides the PPFMs into three clades (A, B, and C) as described previously (Green and Ardley, 2018). Clade A (36 species) is the largest clade, containing the type species *Mb. organophilum* and comprising the genus *Methylobacterium*. Clade B consists of nine *Methylorubrum* species, one of which is *Mr. extorquens*, the type species of the genus *Methylorubrum*. Clade C is divided into subclade C1, consisting of 11 species, subclade C2, consisting of two species (*Methylobacterium isbiliense* and *Mb. nodulans*), and *Mb. oryzihabitans*. As described above, the 16S rRNA gene sequences extracted from genome assemblies had problems, we did not use the 16S rRNA tree to infer the phylogenetic relationship; instead, we used whole-genome information as detailed below.

### Average Nucleotide Identity, Digital DNA–DNA Hybridization, and Whole-Genome Phylogeny

Most of the pairwise FastANI values ranged between 78.8 and 92.9%, with an average of 81.7%. Most of the pairwise dDDH values ranged between 21 and 45.8%, with an average of 23.5% (Supplementary Tables 6, 7). A clear correlation was seen between ANI and dDDH values (Supplementary Figure 2). Some combinations showed high ANI and dDDH values of more than 95 and 70%, respectively. *Mb. oryzae* CBMB20^*T*^, *Methylobacterium fujisawaense* DSM 5686^*T*^, and *Methylobacterium phyllosphaerae* CBMB27^*T*^ were genomically close to each other (98.8% ANI, 90.3% dDDH, and 0.03–0.47% GC% difference). Another close genomic relationship was found between *Mr. thiocyanatum* JCM 10893^*T*^ and *Mr. populi* BJ001^*T*^, which had a relatively high ANI of 97.8%, a high dDDH% of 83%, and a low GC% difference of 0.1%.

**FIGURE 1:**
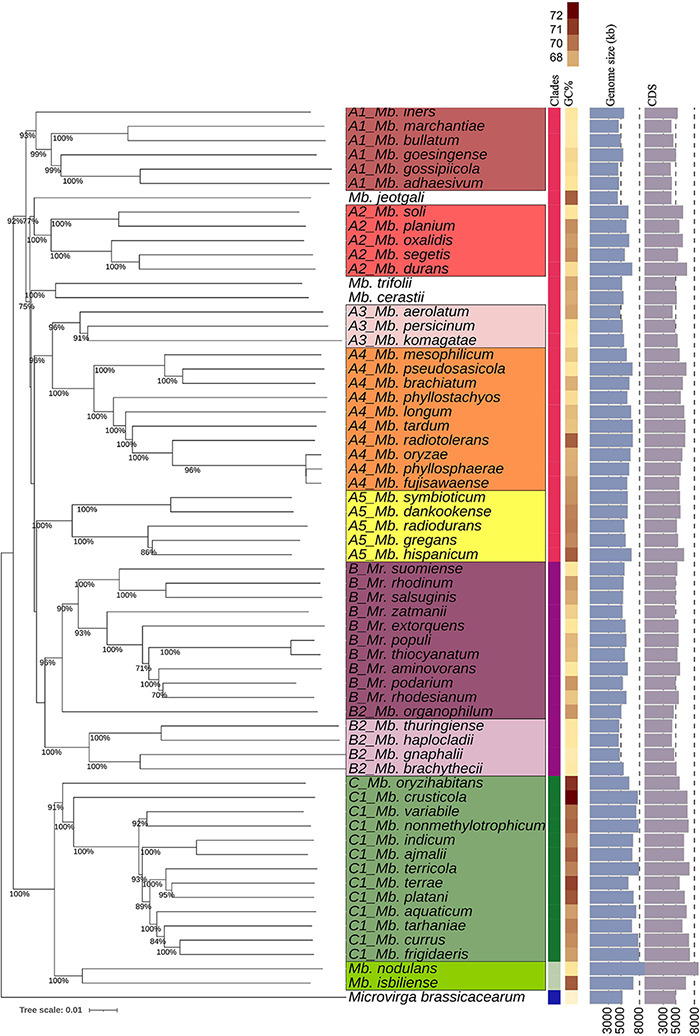
TYGS-genome blast distance phylogeny (GBDP) of 62 PPFM type strains and an outgroup represented by *Microvirga brassicacearum*^*T*^. Colored column bars (left to right) indicate each strain’s clade, genome GC% content, genome size in kb, and number of coding sequences. Branch lengths are scaled based on GBDP formula d5 and numbers above the branch are bootstrap support values from 100 replications.

A whole-genome phylogenetic tree was created with TYGS webtool based on the Genome Blast Distance Phylogeny (GBDP) approach, including *Microvirga brassicacearum* (CDVBN77^*T*^, GCA_008757455.1) as an outgroup. Clade A of *Methylobacterium* represents the largest clade, consisting of five subclades (defined here as A1–A5, Figure 1) and three phylogenetically distinct species (*Methylobacterium jeotgali* LMG 23639^*T*^, *Methylobacterium cerastii* DSM 23679^*T*^, and *Mb. trifolii* DSM 23632, defined here as A). The species within each subclade had relatively high pairwise dDDH and ANI values compared to the other species (Supplementary Tables 6, 7). The GBDP genome tree showed some inconsistency with the 16S rRNA gene tree, especially regarding clade B phylogeny. Unlike the 16S rRNA gene phylogeny, which showed a clear separation between *Methylobacterium* and *Methylorubrum* species, the GBDP tree showed clade B nesting with some *Methylobacterium* species, which we referred to as subclade B2 (Figure 1). This subclade B2 included five *Methylobacterium* species (*Mb. thuringiense* DSM 23674, *Methylobacterium brachythecii* DSM 24105^*T*^, *Methylobacterium haplocladii* DSM24195^*T*^, *Methylobacterium gnaphalii* DSM 24027^*T*^, and *Mb. organophilum* NBRC 15689^*T*^). *Mb. organophilum* NBRC 15689^*T*^, the type species of the genus *Methylobacterium*, was the closest to Clade B. The members of clade C, in contrast, were clustered separately from the other two clades. Clade C had higher GC%, larger genome size, and higher CDS count than the other two clades (Figure 1). GBDP phylogenetic trees are constructed based on the interpretation of pairwise whole-genome similarity, which counts paralogs and other regions within the genomes in addition to orthologous genes (Henz et al., 2005; Meier-Kolthoff and Göker, 2019). After identifying the high score segment pairs (HSPs) with genome-based blastn, a computational algorithm that determines the distances between species, a whole-genome-based phylogeny is created (Henz et al., 2005). This method is widely used to evaluate the taxonomic placements of various species (Meier-Kolthoff et al., 2013; Meier-Kolthoff and Göker, 2019). Therefore, it can be said that our GBDP tree depicts the phylogenetic relationships among the PPFMs with greater precision compared to the tree generated based on the single 16S rRNA gene alone.

### Pan-Genome Analysis

The BPGA pan-genome of 62 PPFM strains already contains 55,128 proteins and is still open, suggesting that the contents of the PPFM genomes are very diverse. The PPFM core genome includes 711 proteins, the accessory genome (containing genes common to two or more strains but not to all strains) includes 2,886–5,500 proteins, and the unique genome (containing genes found exclusively in one strain) includes 147–1,764 genes (Supplementary Table 8). Most of the strains showed low numbers (0–82) of exclusively absent proteins.

### The Pan-Genome and Core Phylogenetic Trees of the Pink-Pigmented Facultative Methylotrophs

**FIGURE 2:**
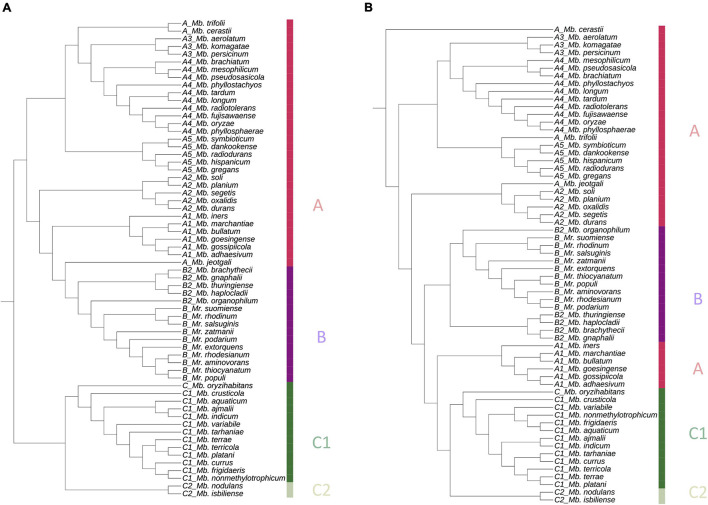
Phylogenetic trees based on **(A)** pan matrix, binary gene presence, and absence matrix (1/0) and **(B)** concatenated sequences of core 711 proteins from the 62 PPFMs. Both trees are generated with BPGA pipeline, and presented with iTOL. The color bar indicates each strain’s clade.

The pan-genome tree created with the presence and absence binary matrix of all proteins was almost consistent with the GBDP tree. Clade A was separated into several subclades, clade B was clustered together with subclade B2, and clade C members were clustered distinctly (Figure 2A).

Meanwhile, the phylogenetic tree based on 711 core proteins amino acid sequences alignment correlated well with the GBDP tree. The species compositions of subclades A1–A5 were consistent with those in the previous trees (Figure 1). Subclade B2 was clustered with clade B, while clade C was phylogenetically distant from the other clades (Figure 2B).

All trees (Figures 1, 2A,B) show the close phylogeny among *Mb. oryzae* CBMB20^*T*^, *Mb. fujisawaense* DSM 5686^*T*^, and *Mb. phyllosphaerae* CBMB27^*T*^, between *Mr. thiocyanatum* JCM 10893^*T*^ and *Mr. populi* BJ001^*T*^, between *Mb. organophilum* NBRC 15689^*T*^ and clade B, and between the subclades A3 and A4.

### Kyoto Encyclopedia of Genes and Genomes Assignments and Functional Annotation of the Pink-Pigmented Facultative Methylotroph Pan-Genome

KEGG functional annotation and distribution analysis of the core, accessory, and unique proteins is shown in Supplementary Figure 3A. The PPFM genomes, as a whole, are relatively highly enriched in the metabolism process. The core genome was enriched in genetic information processing, while the accessory and unique genomes are enriched in environmental information processing. More detailed information is shown in Supplementary Figure 3B. The core genome is noticeably enriched in the biosynthesis of secondary metabolites and the metabolism of cofactors and vitamins. The accessory and core genomes are enriched in motility. Interestingly, the accessory and unique genomes are enriched in the biodegradation of xenobiotics. Because this type of analysis yields only general information, we investigated further as described below.

### Pathway and Module Analysis

**FIGURE 3:**
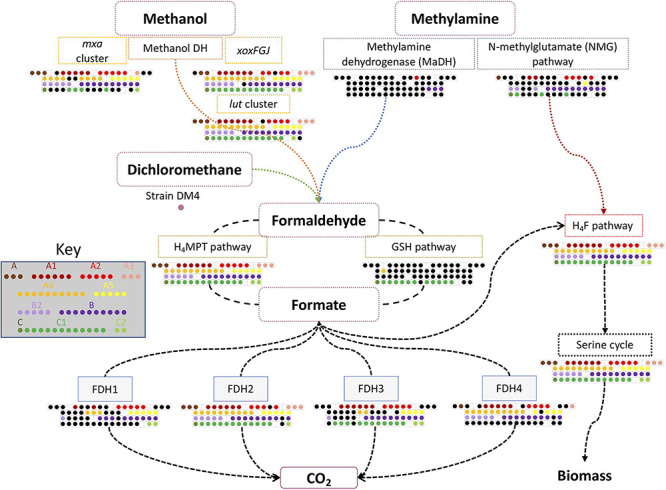
Essential pathways related to C1 metabolism in PPFMs. These pathways include methanol, methylamine, and dichloromethane metabolism. The conservation of gene clusters in each *Methylobacterium* species is indicated by colored dots. *Methylobacterium* type strains are arranged in subclades as shown in Supplementary Table 9. A gene cluster is considered conserved when more than 75% of its genes are present. Black dots indicate a missing cluster or the presence of less than 75% of the gene cluster.

**FIGURE 4:**
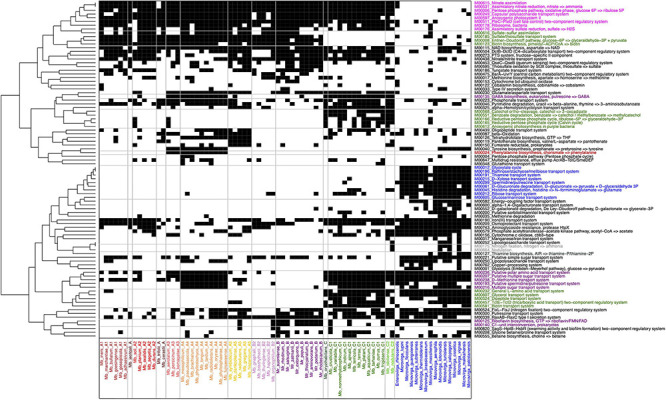
The presence and absence of functional modules and pathways found in PPFMs and the other *Methylobacteriaceae* strains analyzed using the Genomaple pipeline. The presence and absence matrix (1/0) is presented in black and white, respectively. KEGG modules ID and pathways names are shown on the right. Colors: pink, specific to PPFM species; blue, specific for *Microvirga* spp.; red, specific to A3 and A4; purple, specific to B, B2, and some species from A subclades; green, specific to clade C among PPFMs; and gray, nitrogen fixation and nodulation genes found in *Mb. nodulans* ORS 2060^*T*^.

More precise information on functional pathways and modules was obtained using the Genomaple pipeline (Figure 3). To clarify the PPFM-specific functions, other members of *Methylobacteriaceae* were also included in this analysis. The traits that most clearly distinguish the other *Methylobacteriaceae* from the PPFMs are the absence of a nitrate reduction and assimilation module, anoxygenic photosystem II, sulfate reduction and assimilation, and a capsular polysaccharide transport system, and the presence of a glyoxylate cycle, degradation systems for D-glucuronate and histidine, and transport systems for D-xylose, raffinose/glucose/mannose, thiamine, and spermidine/putrescine (Figure 4).

Distinctively from the other PPFMs, clade C lacks modules for a sulfate/thiosulfate transport and assimilation system, an Entner-Doudoroff pathway, and biotin synthesis, but carries modules for transporters for, L-amino acid, glycerol, tricarboxylic acid, dipeptide, and biotin, in addition to a reductive pentose phosphate cycle, anoxygenic photosynthesis (purple bacteria), and degradation pathways for catechol and benzoate. Clade B does not exclusively lack or possess any modules that distinguish it from the members of clade A, with one exception: only clade B possesses a C1-unit interconversion and riboflavin biosynthesis pathway. Together with the members of subclades B2 and A2, clade B members lack transport systems for D-methionine and sugars, and pathways for GABA biosynthesis and pyrimidine degradation. Subclades A3 and A4 exclusively possess the capacity for phenylalanine biosynthesis. It is worth noting that the nitrogen fixation module is found only in *Mb. nodulans* ORS 2060^*T*^ and some members of *Microvirga*.

### Methylotrophy Gene Clusters

Although Genomaple analysis indicated that the “C1-unit interconversion” module is conserved only in clade B, this cannot be true given that most of the PPFMs are methylotrophic. Therefore, we investigated the genes for the C1 assimilation pathway manually. The genes involved in methylotrophy (Supplementary Table 2) were extracted from model strains of *Mr. extorquens* AM1, *Mr. extorquens* PA1, *dichloromethanicum* subsp. *dichloromethanicum* DM4, and *Mb. aquaticum* MA-22A. Cluster completeness was also calculated based on the total number of genes present in each cluster and recorded as a percentage (Supplementary Table 9). The detailed data are presented in Supplementary Table 10. A simplified depiction of the presence of all clusters is provided in Figure 3.

Methylamine utilization genes were examined with methylamine dehydrogenase (MaDH) cluster of *Mr. extorquens* AM1 and the N-methylglutamate (NMG) pathway of *Mr. extorquens* PA1 as queries (Nayak and Marx, 2014). Only one strain from clade A (*Methylobacterium oxalidis* DSM 24028^*T*^) and three clade B species (*Mr. rhodesianum* DSM 5687^*T*^, *Methylorubrum podarium* DSM 15083^*T*^, and *Methylorubrum aminovorans* NBRC 15686^*T*^) have the whole MaDH gene cluster. The complete NMG pathway genes are conserved in seven species of clade A, nine species of clade B species, three species of subclade B2, and only two species of clade C (*Mb. oryzihabitans* TER-1^*T*^ and *Mb. ajmalii* IF7SW-B2^*T*^). Four other species (*Methylobacterium marchantiae* DSM 21328^*T*^ and *Mr. salsuginis* CGMCC 1.6474^*T*^, *Mb. crusticola* KCTC 52305^*T*^, *Mb. aquaticum* DSM 16371^*T*^) were missing only one gene from NMG pathway. This result may indicate that the NMG pathway is more abundant than the MaDH pathway in PPFMs. Previous studies have pointed out that the NMG pathway is highly abundant not only in methylotrophic bacteria (Kröber et al., 2021a) but can also be found in non-methylotrophic bacteria, which rely on it as a nitrogen source (Chen et al., 2010). In a metagenomic study on soybean and rice symbionts, the NMG pathway was more abundantly detected than the *mau* cluster in *Methylobacterium* (Minami et al., 2016).

The Ca^2+^-dependent MDH gene cluster (*mxa* cluster) consisting of 14 genes was found to be conserved in 27 out of 32 species in clade A (A and A1–A5), all of which have all of the essential genes in the cluster (Figure 3). The remaining five type species (*Methylobacterium tardum* NBRC 103632^*T*^, *Methylobacterium longum* DSM 23933^*T*^, *Methylobacterium persicinum* NBRC 103628^*T*^, *Methylobacterium komagatae* DSM 19563^*T*^, and *Methylobacterium iners* DSM 19015^*T*^) lack *mxa* genes; this absence has no correlation with subclade assignment. All of the type strains in clades B and B2 have the conserved *mxa* cluster except for *Mr. thiocyanatum* JCM 10893^*T*^, in which a gene involved in Ca^2+^ insertion into MxaF (*mxaK*) is missing, yet this strain can grow on methanol in the absence of La^3+^ (shown below). In clade C, *mxaFI* encoding Mxa-MDH subunits were found only in five strains of clade C1 (*Mb. oryzihabitans* TER-1^*T*^, *Methylobacterium indicum* SE2.11^*T*^, *Mb. ajmalii* IF7SW-B2^*T*^, *Methylobacterium tarhaniae* DSM 25844^*T*^, and *Mb. terricola* 17Sr1-39^*T*^) and in one strain of clade C2 (*Mb. nodulans* ORS 2060^*T*^). *Mb. aquaticum* DSM 16371^*T*^ has *mxaF* with an identity of 90%, but is missing many of the other *mxa* genes. The strain AM1 genome encodes two genes/regions for Ln^3+^-dependent XoxF-MDH: the *xoxFGJ* cluster and *xoxF2* (Skovran et al., 2011; Vu et al., 2016). These have 87% amino acid sequence identity with each other and 50% identity to the MxaF of the same strain. To differentiate among *xoxF*-homologs in PPFM genomes, we manually checked whether *xoxF1* was within a cluster with *xoxG* and *xoxJ*. This is the case in 59 of the 62 PPFM strains; the exceptions are *Mb. jeotgali* LMG 23639^*T*^, *Mb. oxalidis* DSM 24028^*T*^, and *Methylorubrum suomiense* DSM 14458^*T*^. In clade A, *Mb. jeotgali* LMG 23639^*T*^ *xoxF1* (AOPFMNJM_03121), whose identity to AM1 *xoxF1* and *xoxF2* is 93 and 89%, respectively, is not arranged within the same cluster as *xoxG* and *xoxJ*. *Mb. oxalidis* DSM 24028^*T*^ has an *xoxF*-like gene (LDDCCGHA_05356) with low identity of 46.9 and 47.3% to AM1 *xoxF1* and *xoxF2*, respectively; in addition, its *xoxG* and *xoxJ* are distantly encoded. In clade B, *Mr. suomiense* DSM 14458^*T*^ has an *xoxF* homolog encoded with *xoxG* and *xoxJ*, but its *xoxF* is a pseudogene encoding a short polypeptide of only 63 amino acids. Interestingly, the *xoxF*s in clade C1 (with the sole exception of *Mb. oryzihabitans* TER-1^*T*^) have higher identity to AM1 *xoxF2* than to AM1 *xoxF1*. We also found that some species possess two highly homologous types of XoxF, which is discussed in detail below.

The Ln^3+^ uptake and utilization (*lut*) cluster was recently identified in strains PA1 and AM1. It plays an important role in Ln^3+^ uptake and the regulation of XoxF1 in the presence of Ln^3+^ (Ochsner et al., 2019; Roszczenko-Jasińska et al., 2020). The cluster consists of genes encoding the ABC transporter, TonB-dependent receptor, and the Ln^3+^-binding protein lanmodulin (LanM). The analysis showed that almost all *lut* genes except for *lutH* (TonB-dependent receptor) are conserved in all PPFM genomes. LutH homologs with an identity greater than 50% with AM1 LutH are exclusively present in clade B members and two strains from clade A (*Methylobacterium bullatum* DSM 21893^*T*^ and *Mb. marchantiae* DSM 21328^*T*^). *lutH* is reported to be necessary for *xoxF*-dependent methylotrophic growth in strain AM1 (Roszczenko-Jasińska et al., 2020). The strains that do not harbor *lutH* may utilize another TonB-dependent receptor.

Dichloromethane utilization genes (*dmcR* and *dmcA*) are exclusively found only in the *Methylobacterium dichloromethanicum* subsp. *dichloromethanicum* DM4 genome. These genes are flanked by IS256 family transposase genes in its chromosome, suggesting that the cluster is mobile among bacterial genomes.

The formaldehyde assimilation pathway was also investigated. The H_4_MPT pathway genes are conserved in all PPFMs. The glutathione-dependent formaldehyde dehydrogenase pathway genes (*gfa, hgd*, and *gfh*) were found mostly in members of clade C and in some members of clade A, but were not found in any members of clade B.

Strain AM1 harbors four formate dehydrogenases (FDH1 to FDH4). Most of the clade B strains and many of the subclade A2 and A5 strains conserve these four modules. Most of the clade C strains lack the FDH1 and FDH4 clusters, while about half of the clade A strains lack the FDH1 and FDH3 clusters.

The H_4_F, folate synthesis, serine cycle, and ethylmalonyl-CoA pathways are indispensable for methanol dissimilation in PPFMs; accordingly, the genes for these pathways are all well conserved. The recently discovered factors TtmR and EfgA, which are involved in methylotrophy regulation and formaldehyde binding, respectively, in strain PA1 (Bazurto et al., 2020, 2021) are also well conserved, but the latter is missing in most clade C strains (Supplementary Table 10). Regarding the other species in the *Methylobacteriaceae* family, *Microvirga massiliensis* JC119T^*T*^, an isolate from human feces (Caputo et al., 2016), has some of the methylotrophy gene clusters. The complete *xoxFGJ* cluster and two of the GSH pathway genes are also present in the genomes of some *Microvirga* species. Their methylotrophy should be experimentally verified.

### Methanol Dehydrogenase and Alcohol Dehydrogenase Homologs

The MDHs (MxaF and XoxF) belong to a larger PQQ-dependent periplasmic alcohol dehydrogenase (ADH) family. The members of this PQQ-ADH family can be separated into three types. Type I contains MDHs (XoxF and MxaF), type II contains quinohemoproteins, and type III contains ADHs that are only found in acetic acid bacteria. Type I and II ADHs can be separated into MDHs and a broad category of others (PQQ-ADH types 1–9) (Keltjens et al., 2014). XoxF-type MDHs are encoded widely in many bacterial genomes and can be separated phylogenetically into five families known as XoxF1–XoxF5 (Chistoserdova, 2011). XoxFs from alpha-, beta-, and gamma-proteobacteria mainly belong to the XoxF1, XoxF3, and XoxF5 families (Chistoserdova, 2011; Huang et al., 2018). The XoxF5 family is the most abundant of these. Multiple genes for ADHs coexist in the PPFM genomes. *Mr. extorquens* AM1, for example, contains *xoxF1* and *xoxF2*, both of which belong to the XoxF5 family. Another example is ExaF, an Ln^3+^-dependent ethanol dehydrogenase (Good et al., 2016).

**FIGURE 5:**
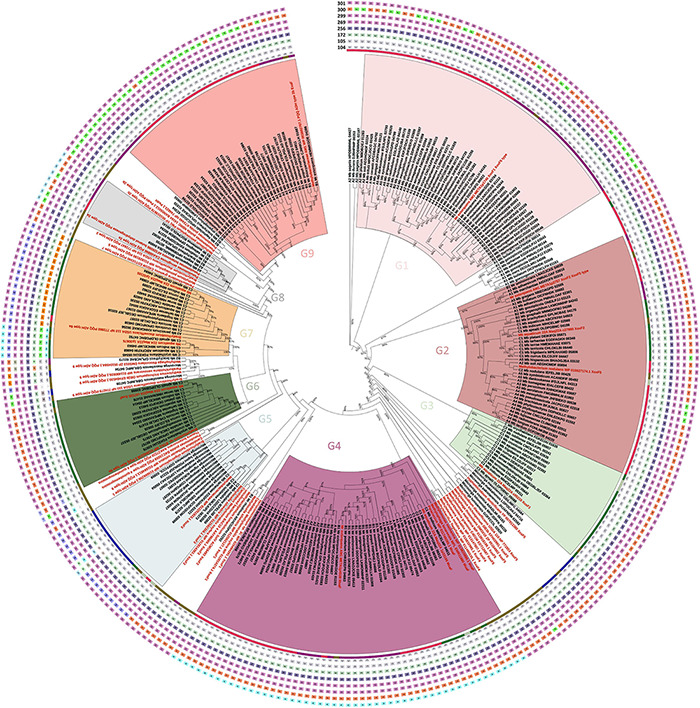
Maximum likelihood phylogeny of PQQ-ADH homologs found in *Methylobacteriaceae* genomes. The tree was built based on the alignment of 252 homologs and 49 reference sequences. The tree was created with FastTree v2.1, and the local support values (>0.7) on the branches shown as a percentage. The reference sequences are shown in red. The highlighted clades labeled G1–G9 represent the following groups: G1, XoxF5-type MDH (XoxF1 in AM1); G2, XoxF5-type MDH (XoxF2 in AM1); G3, XoxF5-type MDH (XoxF1 in 22A); G4, MxaF-type MDH; G5, XoxF3-type MDH; G6, ExaF-type ADH (ExaF in 22A); G7, PQQ-ADH type 6a; G8, PQQ-ADH type 2a; and G9, PQQ-ADH type 2b (ExaF in AM1). The inner colored circle around the tree indicates different PPFM clades (A,B,B2,C,C1, and C2, same as Figure 2) and *Microvirga* species (blue). The outer circles indicate different amino acid residues that are important for the coordination of metals (Ca^2+^ or Ln^3+^) in the active sites of PQQ-ADHs. Each abbreviation of amino acid is shown in a different colored box. The positions of amino acid residues corresponding to the mature XoxF from *Methylacidiphilum fumariolicum* SolV (C104, C105, E172, N256, D269, D299, Y300, and D301) are presented in the outer circles from the inside to the outside in that order.

Each PPFM genome encodes between two and seven PQQ-ADH and MDH orthologs (Figure 5). Our phylogenetic analysis revealed that these orthologs could be divided into nine major (G1–G9) and several minor groups. The first group (G1), represented by the well-characterized XoxF1 from strain *Mr. extorquens* AM1, is present in members of clades A and B. Of note, many genomes encode duplicated genes of this group. The second group (G2), represented by the XoxF2 from strain AM1, is found mostly in clades A and C. The third group (G3) contains XoxF1 from *Mb. aquaticum* strain 22A, an Ln^3+^-dependent MDH that also participates in the regulation of *mxaF* in this strain (Masuda et al., 2018; Yanpirat et al., 2020). This group is found distinctively in clade C members. Most XoxF-type MDHs present in the PPFMs are from the XoxF5 family. The sequences within these groups conserve Asp^301^, whose position corresponds to that of the mature XoxF polypeptide from *Methylacidiphilum fumarioricum* SolV Mfum_190005, (Pol et al., 2014), which determines the metal specificity of XoxF proteins to Ln^3+^. Group 4 contains MxaF from the strain AM1 as a representative as well as orthologs from clades A and B; it is also found in some members of clade C. This group conserves Ala^301^, which determines the metal specificity of MxaF proteins to Ca^2+^ (Pol et al., 2014). Group 5 (the XoxF3 family) consists mainly of orthologs from the *Microvirga* species and members of clade C2, but also contains the only XoxF found in *Mb. oxalidis* DSM 24028^*T*^. This group differs from the XoxF5 family in that it has the amino acid Trp^300^ rather than Tyr^300^ or Phe^300^. Group 6 is found mostly in clade C1 members and contains a homolog of ExaF found in *Mb. aquaticum* strain 22A (Yanpirat et al., 2020). Group 7 includes PQQ-ADH type 6a and is found mostly in members of clade C1. Group 8 contains PQQ-ADH type 2a, a calcium-dependent ADH (Keltjens et al., 2014), and consists of a small group found in members of clades A and B. This group has a conserved Ser^301^. Group 9 includes ExaF from *Mr. extorquens* strain AM1 and several orthologs from members of clades A and B. The characteristic double cysteine (CC, positions 131–132) in the MDH of *Methylococcus capsulatus* (Bath) has recently been shown to be important for its catalytic activity (Chan et al., 2021). This CC is conserved in almost all orthologs, except for some minor members of PQQ-ADH groups 7, 8, and 9.

Thus, the multiple copies of PQQ-ADH orthologs that are encoded in different PPFM clades are phylogenetically different. The presence of multiple copies may suggest the importance of these genes for the species’ survival in and adaptation to different environments. This analysis also showed that there are some minor groups that have never been characterized in terms of their substrate specificity, induction, and metal requirements. Most XoxFs found in the PPFMs are from the XoxF5 family. The XoxF3 family, in contrast, is underrepresented. *Methylorubrum rhodinum* DSM 2163^*T*^ is the only strain to possess a member of the XoxF1 family. XoxFs from other species within the *Methylobacteriaceae* family (i.e., the *Microvirga* species) are mainly members of the XoxF1 and XoxF5 family. The XoxF4 family was not present in the PPFMs; this is in keeping with the fact that XoxF4 is abundant only in Betaproteobacteria (Huang et al., 2018). Although the amino acid Asp^301^ is supposed to determine specificity to Ln^3+^ (Pol et al., 2014), it is worth mentioning that, in a special case, Ca^2+^ was found in the purified *xoxF*-type MDH from *Candidatus* Methylomirabilis oxyfera (Wu et al., 2015).

### Secondary Metabolite Clusters

Secondary metabolite clusters were identified using the antiSMASH pipeline (Supplementary Table 11). The most abundant cluster in PPFM genomes is the terpene cluster. All genomes have two or more terpene clusters, one of which is a carotenoid cluster, while the others are unknown or malleobactin synthesis clusters with low similarity value. The homoserine lactone cluster is found in most genomes, and members of clade C are particularly likely to have two or more clusters. Type I polyketide synthase, non-alpha poly-amino acids like e-polylysine, and redox-cofactor (PQQ) clusters are also present in almost all genomes. Some strains have non-ribosomal peptide synthetase clusters (NRPS) and NRPS-like clusters. Compared to PPFMs in other clades, clade C PPFMs tend to contain relatively larger numbers of clusters for various secondary metabolites.

### Plant Growth-Promoting Genes

Pink-pigmented facultative methylotrophs can induce plant growth in a direct way by producing phytohormones such as auxin and cytokinin. The complete L-tryptophan pathway for IAA biosynthesis found in *Mb. nodulans* ORS 2060^*T*^ (L-tryptophan decarboxylase, monoamine oxidase, and aldehyde dehydrogenase) is also found mostly in members of clade C and in some members of clade A (Supplementary Table 12). Monoamine oxidase and aldehyde dehydrogenase found in *Mb. oryzae* CBMB20^*T*^ were also found in subclades A3 and A4 and clade C. The tRNA delta(2)-isopentenylpyrophosphate transferase gene (*miaA*), which is responsible for cytokinin synthesis through the *trans*-zeatin pathway (Koenig et al., 2002), is conserved in almost all PPFM genomes. Bacterial 1-aminocyclopropane-1-carboxylic acid (ACC) deaminase (AcdS) is involved in lowering ethylene levels in plants. The importance of bacterial D-cysteine desulfhydrase (DcyD), which is structurally homologous to ACC deaminase, is unknown, but it may be involved in the production of hydrogen sulfide, which also stimulates plant growth (Ekimova et al., 2018). Our analysis showed that *acdS* was found in clades A and C but not in clade B. *dcyD* was found mostly in members of clades B and B2 and in a few members of clade A but not in clade C. The chitinase gene was found in a few strains in clade A, while the pectinase gene was found in a few strains in clade B. A number of antioxidant enzymes (glutathione peroxidase and glutathione oxidase) were found to be conserved in the genomes of all PPFMs. Ergothioneine is an unusual amino acid involved in UV resistance in *Methylobacterium* (Alamgir et al., 2015). The genes for ergothioneine synthesis are conserved across all PPFMs (Supplementary Table 12).

### Phenotyping of Pink-Pigmented Facultative Methylotrophs

#### Pink-Pigmented Facultative Methylotroph Growth Assay on Single Carbon Sources

To examine the correlation between the PPFMs’ genotypic data and their phenotypes, growth tests were performed under various conditions (Supplementary Table 13). Growth on methanol (in the absence of Ln^3+^) has been regarded as a hallmark of PPFMs, although some strains have been known to be unable to grow on methanol. The species in clade A that are lacking *mxaF* (*Mb. tardum* NBRC 103632^*T*^, *Mb. longum* DSM 23933^*T*^, *Mb. persicinum* NBRC 103628^*T*^, *Mb. komagatae* NBRC 103627^*T*^, and *Mb. iners* DSM 19015^*T*^) did not grow on methanol. All species in clade B, however, were able to grow well on methanol. Some species in clade C1 did not grow or showed very weak growth on methanol. These species lack the whole *mxa* cluster as described above, although some of them have been reported to grow on methanol (Park et al., 2018; Kim et al., 2019; Jia et al., 2020). *Mb. cerastii* DSM 23679*^*T*^* also did not grow on methanol, although it has the whole *mxa* cluster; this strain showed very weak growth in various tests throughout the whole analysis.

As noted already, most strains have XoxF type MDH that is activated by Ln^3+^ (Fitriyanto et al., 2011; Hibi et al., 2011; Pol et al., 2014). When tested on methanol in the presence of La^3+^, almost all strains, including those that showed weak or no growth in the absence of La^3+^, were able to grow. *Methylobacterium nonmethylotrophicum* 6HR-1^*T*^ has been reported as a non-methylotrophic bacterium lacking the *mxa* cluster (Feng et al., 2020), but the strain *Mb. nonmethylotrophicum* KCTC 42760^*T*^ is indeed an Ln^3+^-dependent methylotrophic strain. *Mb. tardum* NBRC 103632^*T*^, *Mb. longum* DSM 23933^*T*^, *Mb. persicinum* NBRC 103628^*T*^, *Mb. komagatae* NBRC 103627^*T*^, *Mb. iners* DSM 19015^*T*^, *Methylobacterium variabile* DSM 16961^*T*^, and *Methylobacterium platani* JCM 14648^*T*^ require La^3+^ to grow on methanol. These results indicate that Ln^3+^-dependent methylotrophy is of primary importance compared to Ca^2+^- and MxaF-dependent methylotrophy in PPFMs. Yet the conservation of the latter pathway in many PPFMs suggests that they are also adaptable to environments where Ln^3+^ is not readily available, such as the phyllosphere.

We found that 11 strains in clade A can utilize methylamine and that all of these have the NMG pathway, although *Mb. oxalidis* DSM 24028^*T*^ has both the MaDH pathway and the NMG pathway. All clade B strains grew on methylamine, owing to their NMG genes rather than to *mau* clusters. Most clade C1 and C2 strains were not able to utilize methylamine. This result is almost entirely consistent with a previous description of the three clades (Green and Ardley, 2018), except that a number of strains from clade A are able to use methylamine. The *mau* (methylamine utilization) gene cluster in strain AM1 is flanked by the IS elements *ISMex15* and IS*Mdi3*, suggesting that the cluster is mobile among bacterial genomes, as are the other metabolic genes for C1 compounds including chloromethane and dichloromethane.

*Methylobacterium dichloromethanicum* subsp. *dichloromethanicum* strain DM4 was the only strain capable of utilizing dichloromethane.

#### Pink-Pigmented Facultative Methylotroph Growth Assays on Different Carbon Sources

Almost all tested strains grew well on ethanol irrespective of the presence of La^3+^; many C1 strains, however, showed weak growth in the absence of La^3+^. The distribution of sugar utilization in clade A is almost random, while all clade B strains other than *Mr. extorquens* strains as well as almost all clade C1 strains grew well on the tested sugars. Glycerol could support the growth of most strains. Almost all of the strains grew well on PDA medium. Most of the strains from clades B and C grew on NB medium, while strains from clade A and subclade B2 showed weaker growth under these conditions (Supplementary Table 13). Strains from subclade A4 did not grow on sucrose.

#### Growth Under Different Stress Conditions

All species were tested for growth in 1% NaCl and in the presence or absence of ampicillin (25 or 50 mg/l). In clade A, ability to grow under each of these three conditions did not correlate with membership in subclades A1 and A2, but did correlate with membership in subclades A3, A4, and A5 and with successful growth on R2A medium regardless of ampicillin and NaCl treatment. All species in clade B tolerated NaCl. Interestingly, the species in clades C1 and C2 were not able to grow under 1% NaCl conditions, but most of them showed resistance to ampicillin (Supplementary Table 13).

#### API 20NE Test Result

Phenotypic analysis was performed by means of the API 20NE test. After 24 h of incubation, nitrate reduction, indole production, and glucose fermentation were recorded. In general, as shown in Supplementary Table 14, all PPFM strains were negative for indole production and glucose fermentation. Nine species out of the 60 tested were positive for reduction of nitrates to nitrites; these were mostly members of clades A, B, and C2. All species were negative for β-glucosidase, protease, β-galactosidase, p-nitrophenyl-β-D-galactopyranosidase, and assimilation of capric acid. Most of the strains were urease-positive, but noticeably weak urease production was detected for some members of clade C1. On the other hand, there was a clear variation regarding the assimilation of sugars and organic acids. The majority of species in subclades A1, A2, and A3 were positive for sugar assimilation but weak or negative for the assimilation of organic acids. Most strains from subclades A4 and A5 were positive for the assimilation of all sugars and organic acids except for adipic acid. Some clade B strains showed weak assimilation of sugars, but most strains were positive for the assimilation of N-acetyl-glucosamine and organic acids. In the subclade B2, some members showed weak growth on both tested sugars and organic acids. Most strains in clade C1 were positive for the assimilation of sugars. Clade C2 members were negative or weak for most of the tests.

Some species (*Mb. iners* DSM 19015^*T*^, *Mb. thuringiense* DSM 23674, *Mb. trifolii* DSM 23632, *Mb. cerastii* DSM 23679^*T*^, and *Mb. crusticola* KCTC 52305^*T*^) were negative for most of the tests, consistently showing weak growth although they belong to different subclades.

### Indole Acetic Acid Production Through the L-Tryptophan Pathway

Most of the PPFMs produced IAA at concentrations ranging from 8 to 29 μg/ml; in contrast, the control pathogenic bacteria *Pseudomonas syringae* DC 3000 produced high concentrations of IAA (57 μg/ml, data not shown) (Supplementary Table 13). The members of clade A (especially *Mb. gnaphalii* DSM 24027^*T*^, *Mb. brachythecii* DSM 24105^*T*^, *Mb. oxalidis* DSM 24028^*T*^, and *Mb. dankookense* DSM 22415^*T*^) and clade B were potent producers compared to the members of clade C, although clade C species have the complete L-tryptophan pathway. This result correlates with the fact that PPFMs can enhance plant growth directly by the production of phytohormone as one of the important direct PGP mechanisms (Subhaswaraj et al., 2017; Vadivukkarasi and Bhai, 2020), and with the fact that the addition of tryptophan induces IAA production (Omer et al., 2004). Thus, although the tested genomes do not have the whole L-tryptophan pathway of *Mb. nodulans* ORS 2060^*T*^, these strains were nevertheless able to produce IAA. These strains may have genes with an identity lower than 50%, or they may employ a different pathway to produce IAA. The blast hits for L-tryptophan decarboxylase indeed had very low identity values, and most strains were missing it entirely. This is also seen in the *Mb. oryzae* CBMB20^*T*^ genome, which is missing L-tryptophan decarboxylase (Kwak et al., 2014).

### Antifungal Activity

We tested PPFM strains for antifungal activity, which can be an indirect means of promoting plant growth. Subclade A4, which includes *Methylobacterium radiotolerans* IAM 12098^*T*^, *Mb. oryzae* DSM 18207^*T*^, and other closely related strains, showed strong antifungal activity (Supplementary Figure 4); it is worth noting that some of these strains carry chitinase genes. The members of subclade A5 also showed strong antifungal activity. In clade B species, antifungal activity was moderate except in *Mr. rhodesianum* DSM 5687^*T*^ and *Mr. extorquens* NBRC 15687^*T*^ which showed the strongest activity. Most of the strains in clade C1 had a clear suppressive effect on *Fusarium* growth. Our genome mining data showed that chitinase, cellulase, pectinase, and a number of secondary metabolite clusters were conserved across the PPFMs (Supplementary Table 13). Siderophore production and NRPS may be involved in this antifungal activity, but further investigation is needed to identify which secondary metabolite is active against each particular pathogen.

### Rice Cell Elicitation

We employed a rice cell elicitation assay to assess plant cell reactions against PPFMs. As shown in Supplementary Figure 5, none of the PPFMs caused any detectable coloration of rice cells or increased the turbidity of the medium, nor did the negative control. The pathogenic bacteria *P. syringae* DC3000 and *Serratia marcescens* subsp. *marcescens* strains 1–7 and 4–24 (Wari et al., 2019) caused clear brown coloration in rice cells, indicating the activation of secondary metabolite production, and increased turbidity of the media, indicating rapid (in less than 20 h) microbial growth. These results suggest that all of the studied PPFMs are recognized as non-plant-pathogenic bacteria. This is supported by the fact that PPFMs are abundant endophytic and epiphytic bacteria (Delmotte et al., 2009; Knief et al., 2010; Tani et al., 2012; Roodi et al., 2020). A negative effect of rice leaf bleaching by inoculation with a strain of *Mb. indicum* has been reported, however (Lai et al., 2020).

## Discussion

### General Discussion

Pink-pigmented facultative methylotrophs are environmentally important bacteria due to their roles as phyllospheric symbionts and are useful as platforms for fine chemical synthesis using methanol as a cheap feedstock. Their diverse metabolic capacity, enabling their growth on not only methanol but also other C1 compounds, has led to much interest in the genetics and biochemistry of methylotrophy. From a taxonomic point of view, the family *Methylobacteriaceae* contains the genera *Methylobacterium*, *Methylorubrum*, *Microvirga*, *Psychroglaciecola*, and *Enterovirga*. The PPFMs have been classified into three major clades called clades A, B, and C, and the clade B species are currently classified as *Methylorubrum* (Green and Ardley, 2018). The main purpose of creating this new genus *Methylorubrum* was to create a more homogeneous bacterial group within the broadly diverse *Methylobacterium* species. However, a recent large survey has proposed uniting *Methylobacterium* and *Methylorubrum* (Hördt et al., 2020). As many as 63 species that may belong to this new unified taxonomic group have been described, while some other species have been reclassified as strains of existing species, such as *Methylobacterium lusitanum* (*Mb. rhodesianum*), and *Mb. chloromethanicum* and *Mb. dichloromethanicum* (subspecies of *Methylobacterium dichloromethanicum*). *Mb. organophilum* ATCC 27886^*T*^ (Patt et al., 1976) and *Mr. populi* BJ001^*T*^ (Van Aken et al., 2004) have been reported to be able to grow on methane, but no follow-up research has reproduced this growth (Dedysh et al., 2004). The species in clades A and B are relatively phenotypically similar to each other, while the species in clade C tend to be phylogenetically distinct from the other clades. Accordingly, they were proposed as species *incertae sedis*, and further evidence is required to clarify their taxonomic position (Green and Ardley, 2018). Although *Methylobacterium* species are often referred to as PPFMs, some species have been reported to be non-pigmented and/or incapable of methanol utilization. This uncertainty motivated us to clarify the genotypes and phenotypes of all species considered PPFMs. We also set out to compare the PPFMs with the other genera in the same family in order to clarify the common characteristics that are unique to PPFMs.

16S rRNA gene sequences have been used for the delineation of many species–species boundaries. It must be noted, however, that PCR-amplified sequences may differ under different laboratory conditions, which complicates attempts to replicate findings (Kato et al., 2005). Usually, the intragenomic heterogeneity of 16S rRNA genes should be within the species threshold, yet contamination with phenotypically and morphologically similar strains may cause ambiguous identification of sequences. In addition to this PCR problem, there are also problems with the 16S rRNA gene sequences from the PPFM genomes related to copy numbers, contamination, missing genes, or assembly quality, as previously reported (Hördt et al., 2020). Therefore, it is more desirable to employ a phylogenetic analysis based on the whole genome. In this study, we sequenced the genomes of 29 strains to cover all known species in *Methylobacterium/Methylorubrum*. This genome data provided precise and detailed insights into the genomics and phylogeny of these species.

The GBDP tree showed that clades A and B are nested together, while clade C is distinct. Our analysis of our newly sequenced genomes has revealed that clade A can be divided into several more homogeneous subclades, each of which shares high pairwise dDDH and FastANI values; in some cases, the species within each subclade are also highly similar phenotypically. Pairwise dDDH values demonstrated that some species had previously been poorly classified based on their 16S rRNA sequences, as discussed below. Whereas a 16S rRNA tree relies on the identity of only one gene sequence, the more accurate phylogenetic tree of PPFMs that we present here is based on whole-genome sequences. These approaches (GBDP, core and pan phylogeny) yielded compatible results, showing that the two genera (*Methylorubrum* and *Methylobacterium*) are phylogenetically close. One of the closest species to clade B was the type species of the genus *Methylobacterium, Mb. organophilum* NBRC 15689^*T*^.

The utilization of methanol is a strategically important ability for PPFMs in the environment. Methanol emitted from plants is reported to affect the distribution of PPFM species in soil (Macey et al., 2020). Although several PPFM species can utilize other C1 compounds such as methylamine and chlorinated methane, all species have at least one MDH cluster that guarantees that they can thrive on methanol, as the present study confirms. Other methylotrophy gene sets (PQQ synthesis, H_4_MPT pathway, serine cycle, *lut* genes, H_4_F pathway, and EMC pathway) are conserved in most PPFM species but are absent in the other *Methylobacteriaceae* species. Since glyoxylate, which is necessary for the serine cycle, is supplied by the EMC pathway (Schneider et al., 2012), the glyoxylate cycle is missing in the PPFM species, but it is present in the other species in the *Methylobacteriaceae* family. Among the methylamine-utilizing PPFMs, the NMG pathway seems to be preferred; likewise, the NMG pathway is believed to be the major methylamine utilization pathway in soybean-associated *Methylobacterium* (Minami et al., 2016) and other methylotrophic bacteria or non-methylotrophic bacteria (Chen et al., 2010; Kröber et al., 2021a).

Pink-pigmented facultative methylotroph genomes encode several homologs of ADH and MDH proteins. Nine phylogenetic groups were observed. XoxF types were predominant, especially those belonging to the XoxF5 family (Chistoserdova, 2011). Phylogenetically, the ExaF types were divided into two groups (PQQ ADH type 2b and PQQ ADH type 6a), both of which are putative Ln^3+^-containing proteins (Keltjens et al., 2014). A small group of putative calcium-dependent ADH PQQ type 2a proteins is also encoded. Generally, the PPFM genomes encode a variety of ADH and MDH proteins, most of which are Ln^3+^-dependent.

*Methylobacterium* species are highly abundant in and adapted to the phyllosphere, which is a harsh environment with limited nutrients, intense UV light, and dramatic fluctuations in weather conditions (Knief et al., 2010; Minami et al., 2016; Yoshida et al., 2017). Our data show that these species are well prepared for these circumstances, however, as they have genes that enable UV damage repair, antioxidative stress resistance (the ergothioneine and peroxidase genes), and carotenoid synthesis. All *Methylobacterium* species have the full carotenoid cluster except for *Mb. nodulans* and *Mb. jeotgali* (data not shown). Each of these two exceptions has a unique niche: *Mb. nodulans* nodulates in *Crotalaria podocarpa* (Jourand et al., 2004), and *Mb. jeotgali* is found in a Korean salted seafood (Aslam et al., 2007). As neither of these niches requires carotenoid synthesis, some genes encoding this ability have been lost during evolution and adaptation. Although there are therefore some non-pigmented PPFMs, they are the exceptions, whereas most PPFMs are pink-pigmented to varying degrees.

KEGG functional annotation has shown that the core genes of the PPFM species, which are important for their survival and symbiotic interaction with their hosts, are enriched with cofactor and vitamin biosynthesis genes. Some microalgae species are reported to be dependent on *Methylobacterium* for supplementation with vitamins (Krug et al., 2020). This analysis also showed that the core genome is rich in secondary metabolite clusters, as confirmed through antiSMASH analysis. These clusters are valuable for plant growth promotion, solubilization of nutrients, and plant protection (Vadivukkarasi and Bhai, 2020). Nitrate reduction and assimilation are also unique characteristics of *Methylobacterium* species; these abilities provide additional benefits for their hosts (Madhaiyan et al., 2010; Calatrava et al., 2018). The anoxygenic photosystem II, also a unique feature, involves a bacterial chlorophyll gene that clusters close to the carotenoid gene cluster. It enables the PPFMs to gain ATP but not to fix carbon or oxygen (Zervas et al., 2018). PPFMs are not only involved in plant growth promotion and methanol utilization, as a few researchers have pointed out before (Ventorino et al., 2014; Eevers et al., 2015; Li et al., 2020), but also in xenobiotic biodegradation, for which the genes are enriched by unique and accessory genome.

This study found that all of the studied species had at least some PGP traits, i.e., at least some conservation of PGP genes (*miaA*, *acdS*, and *dcyD*), IAA production, and antifungal activity. Members of subclades A4 and A5 and clade C1 showed strong antifungal activity. Most clade A4 and clade A5 species were isolated from plants. In the future, we expect that it will be possible to develop plant-growth promoting, antifungal biocontrol agents containing *Methylobacterium* species. However, it is known that only specific species can be isolated from specific plants (Tani et al., 2015), suggesting species-species specificity. By taking a comparative genome-based approach to studying isolates from plants, we may be able to identify the genes or the factors that determine this specificity.

### Taxonomic Consequences: Synonymous Species, New Combinations, and Emendation of the Genus

Through the use of dDDH and ANI to delineate species boundaries, several synonyms have been found within the genus *Methylobacterium* (Hördt et al., 2020) and the family *Rhizobiales* (Volpiano et al., 2021). Our results support the consideration of *Mb. oryzae* and *Mb. phyllosphaerae* as the same species (Green and Ardley, 2018; Hördt et al., 2020; Kröber et al., 2021a). Here we also propose reclassifying *Mb. phyllosphaerae* (Madhaiyan et al., 2009) and *Mb. oryzae* (Madhaiyan et al., 2007) as later heterotypic synonyms of *Mb. fujisawaense* (Green et al., 1988). In addition, as *Mr. thiocyanatum* (Wood et al., 1998) and *Mr. populi* (Van Aken et al., 2004) have >95% ANI and >80% dDDH, both of which are higher than the species–species separation thresholds, *Mr. populi* is a later heterotypic synonym of *Mr. thiocyanatum*.

*Methylobacterium dichloromethanicum* (Doronina et al., 2000) and *Mb. chloromethanicum* (McDonald et al., 2001) are later heterotypic synonyms of *Mb. extorquens* as reported previously (Kato et al., 2005). Hördt et al. (2020) have recently suggested uniting the genera *Methylorubrum* and *Methylobacterium.* Due to these groups’ high dDDH values (70∼79%), those authors also proposed classifying *Mb. chloromethanicum* as *Mb. dichloromethanicum* subsp. *chloromethanicum*, subsp. nov. and *Mr. extorquens* as *Mb. dichloromethanicum* subsp. *extorquens*, subsp. nov. In a recent announcement of newly validated names (Validation list no. 194, DOI 10.1099/ijsem.0.004244), the name *Mb. dichloromethanicum* subsp. *chloromethanicum* was validated, but *Mb. dichloromethanicum* subsp. *extorquens*, subsp. nov. was not. Instead, the name *Mb. dichloromethanicum* subsp. *dichloromethanicum* was also created automatically according to a taxonomical rule in the same validation list. The fact that the name *Mb. extorquens* (Urakami and Komagata, 1984; Bousfield and Green, 1985) had priority was ignored. Therefore, we propose classifying the names as *Mb. extorquens* subsp. *dichloromethanicum* subsp. nov., *Mb. extorquens* subsp. *chloromethanicum* subsp. nov., and *Mb. extorquens* subsp. *extorquens* subsp. nov.

#### Emendation of the genus *Methylobacterium*

The description is as given before (Patt et al., 1976; emend. Green and Bousfield, 1983) with the following additions. Some strains exhibit Ln^3+^-dependent growth on methanol. The genomic G+C content is 66–72.8%. The type species is *Methylobacterium organophilum* and its approximate genome size is 5.07 Mbp.

#### Description of *Methylobacterium extorquens* subsp. *dichloromethanicum* subsp. nov. comb. nov.

Basonym: *Methylobacterium dichloromethanicum* subsp. *dichloromethanicum* (Doronina et al., 2000) Hördt et al., 2020, *Methylobacterium dichloromethanicum* (Doronina et al., 2000).

M. ex.tor’quens. subsp. dichlo.ro.me.tha’ni.cum (di.chlor’o’meth.an’ic.um. M.L. n. chlor; N.L. neut. n. methanum, methane; L. pref. di-, two; N.L. neut. adj. *dichloromethanicum*, dichloromethane utilizing). The description is as given for *Methylobacterium dichloromethanicum* (Doronina et al., 2000). The type strain is DM4 = VKM B-2191 = DSM 6343 = CIP 106787.

#### Description of *Methylobacterium extorquens* subsp. *chloromethanicum* subsp. nov. comb. nov.

Basonym: *Methylobacterium dichloromethanicum* subsp. *chloromethanicum* (McDonald et al., 2001) Hördt et al., 2020, *Methylobacterium chloromethanicum* (McDonald et al., 2001).

M. ex.tor’quens. subsp. chlo.ro.me.tha’ni.cum (N.L. neut. n. *chloromethanicum*, chloromethane-utilizing). The description is as given for *Methylobacterium chloromethanicum* (McDonald et al., 2001). The type strain is CM4 = VKM B-2223 = NCIMB 13688.

#### Description of *Methylobacterium extorquens* subsp. *extorquens* subsp. nov., comb. nov.

Basonym *Methylorubrum extorquens* (Green and Ardley, 2018), *Methylobacterium extorquens* (Urakami and Komagata, 1984) Bousfield and Green, 1985, *Protomonas extorquens* (ex Bassalik, 1913) Urakami and Komagata, 1984. Automatically created based on Rule 40d and lowering to subspecies rank from *Methylorubrum extorquens* (Green and Ardley, 2018).

M. ex.tor’quens. subsp. ex.tor’quens subsp. nov. (ex.tor’quens. L. part. adj. *extorquens*, twisting out). The description is as given for *Protomonas extorquens* (ex Bassalik, 1913) Urakami and Komagata, 1984; Bousfield and Green, 1985. The type strain is TK 0001 = ATCC 43645 = CCUG 2084 = DSM 1337 = JCM 2802 = NBRC 15687 = VKM B-2064.

## Conclusion

We completed whole-genome information for the type strains of PPFMs by newly sequencing the type strains of 29 species in this study. The genome sequence of the type species of the genus *Methylobacterium*, *Mb. organophilum* NBRC 15689^*T*^, is announced in this study. Using whole-genome data, we were able to classify the 62 PPFM species into eight homogeneous groups. Species from *Methylobacterium* and *Methylorubrum* were nested together, indicating the importance of reconsidering their separation into two genera. Only one of the eight groups (Clade C) was uniquely separated from all other species; the members of this clade are also phenotypically different from the others. Methylotrophy gene clusters were one of the unique features common to the PPFMs as opposed to the other genera in the *Methylobacteriaceae*. All PPFM genomes had at least one cluster of methanol dehydrogenase (MDH), and Ln^3+^-dependent MDH was conserved in almost all species. This discovery was verified by phenotyping, which revealed that almost all of the PPFMs were able to grow on methanol with La^3+^ supplementation. These findings highlight the importance of Ln^3+^ as one of the main environmental factors affecting PPFMs. Screening for other factors related to plant symbiosis revealed that all PPFMs produce IAA and trigger no immune response in rice, and that some can inhibit pathogenic fungal growth *in vivo*.

## Data Availability Statement

The datasets presented in this study can be found in online repositories. The names of the repository/repositories and accession number(s) can be found in the article/Supplementary Material.

## Author Contributions

OA, NS, and AT designed the research and drafted the manuscript. OA and YF performed the experiments. OA, YO, HT, TH, NS, and AT performed the data analysis. All authors reviewed the manuscript.

## Conflict of Interest

The authors declare that the research was conducted in the absence of any commercial or financial relationships that could be construed as a potential conflict of interest.

## Publisher’s Note

All claims expressed in this article are solely those of the authors and do not necessarily represent those of their affiliated organizations, or those of the publisher, the editors and the reviewers. Any product that may be evaluated in this article, or claim that may be made by its manufacturer, is not guaranteed or endorsed by the publisher.

## Footnotes

1
https://lpsn.dsmz.de/genus/methylobacterium

2
https://usegalaxy.org

3
https://www.ezbiocloud.net

4
https://tygs.dsmz.de/

5
https://lpsn.dsmz.de

## Supplementary Material

Supplementary Figure 1 TYGS- 16S rRNA tree. This tree was created from the 16S rRNA gene sequences extracted from the assemblies. The tree shows 59 PPFM species and an outgroup represented by *Microvirga brassicacearum*^*T*^.

Supplementary Figure 2 The correlation of pairwise PCR-derived 16S rRNA gene identity, digital DNA-DNA hybridization (dDDH), and average nucleotide identity (ANI) values among all combinations of PPFMs. **(A)** The correlation between dDDH and ANI, **(B)** the correlation between 16S rRNA and ANI, and **(C)** the correlation between 16S rRNA and dDDH. The lower panels show plots of species combinations with high correlation values.

Supplementary Figure 3 Functional annotation and distribution of core, accessory, and unique genes of the PPFMs within KEGG main groups. **(A)** KEGG general category, and **(B)** more specific details of KEGG category. The gene count in each category is shown as a percentage of the total count in each group (core, accessory, and unique). ^∗^The overview category includes carbon metabolism (ko01200), oxocarboxylic acid metabolism (ko01210), fatty acid metabolism (ko01212), degradation of aromatic compounds (ko01220), and biosynthesis of amino acids (ko01230).

Supplementary Figure 4 Antifungal activity of PPFMs against *Fusarium oxysporum*. Among the tested strains, only those with strong inhibition activity are shown. Each PPFM was inoculated onto half of a PDA plate, and the fungus was allowed to grow from the center. The plates were incubated at 25°C for 7–10 days.

Supplementary Figure 5 Rice cell elicitation assay. The PPFMs were inoculated into rice cell suspension, then incubated for 24 h. The numbers represent the tested species listed in Supplementary Table 13. DC3000, *Pseudomonas syringae* DC3000; 1–7 and 4–24, *Serratia marcescens* subsp. *marcescens* isolates 1–7 and 4–24; GlcNAc, chitin oligomer (10 nM); and NC, negative control (saline solution).
