# Supplementary figures and images for: Comprehensive Comparative Genomics and Phenotyping of Methylobacterium Species
Source: Front Microbiol. 2021 Oct 6;12:740610. doi: 10.3389/fmicb.2021.740610 (PMC8561711; doi:10.3389/fmicb.2021.740610)

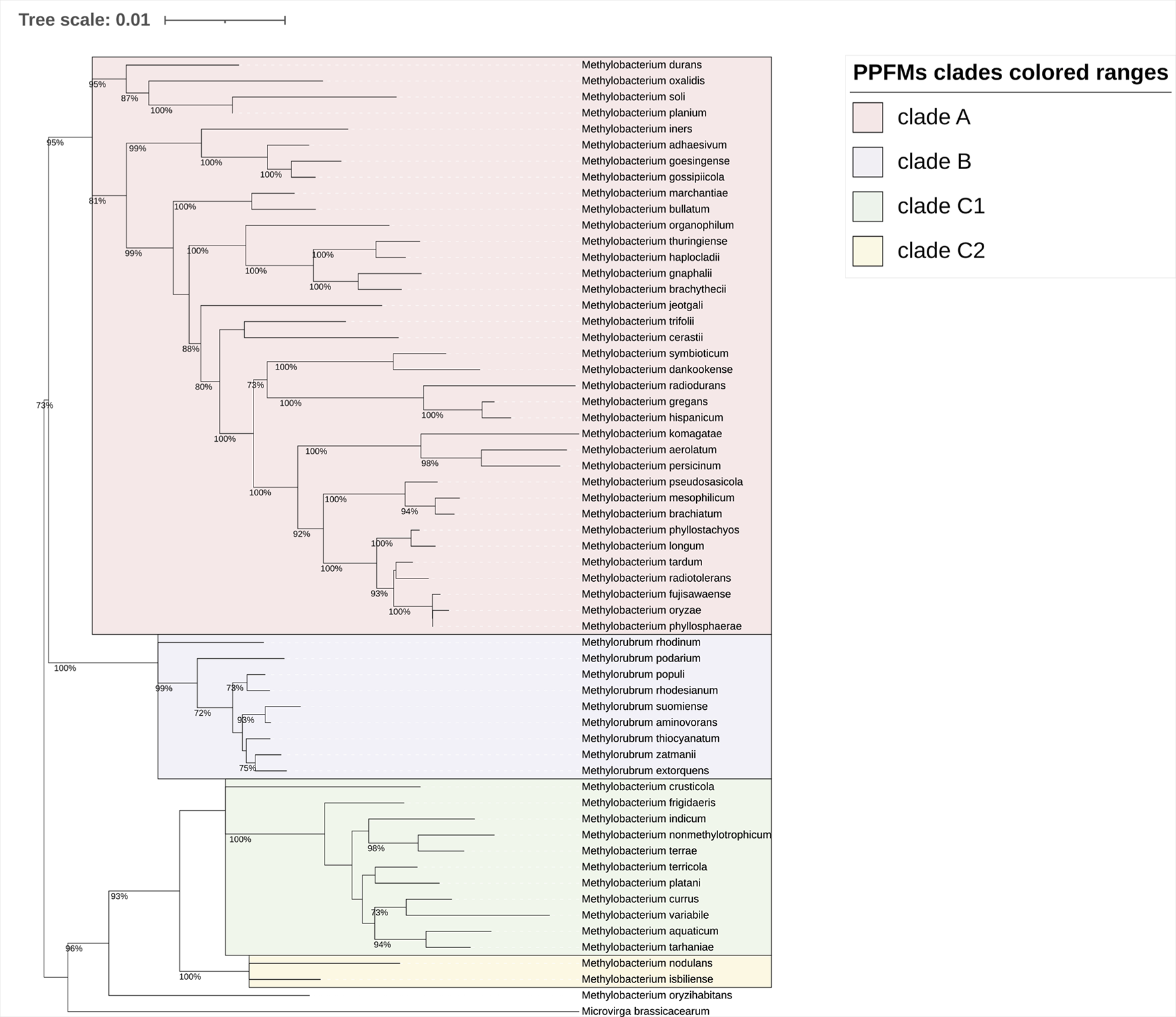

Supplement: Supplementary Figure 1 — TYGS- 16S rRNA tree. This tree was created from the 16S rRNA gene sequences extracted from the assemblies. The tree shows 59 PPFM species and an outgroup represented by Microvirga brassicacearumT. [file Image_1.TIFF]

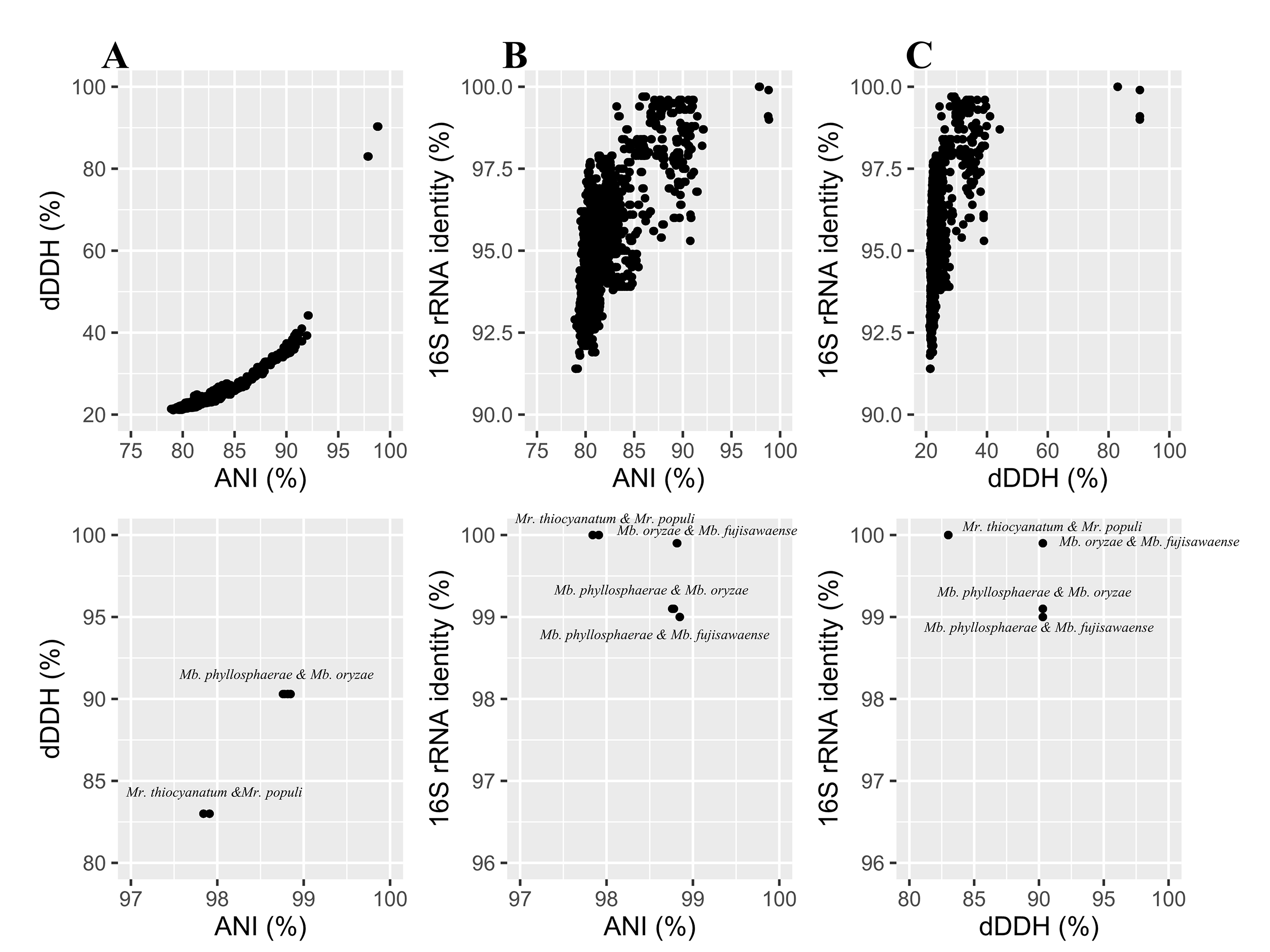

Supplement: Supplementary Figure 2 — The correlation of pairwise PCR-derived 16S rRNA gene identity, digital DNA-DNA hybridization (dDDH), and average nucleotide identity (ANI) values among all combinations of PPFMs. (A) The correlation between dDDH and ANI, (B) the correlation between 16S rRNA and ANI, and (C) the correlation between 16S rRNA and dDDH. The lower panels show plots of species combinations with high correlation values. [file Image_2.tif]

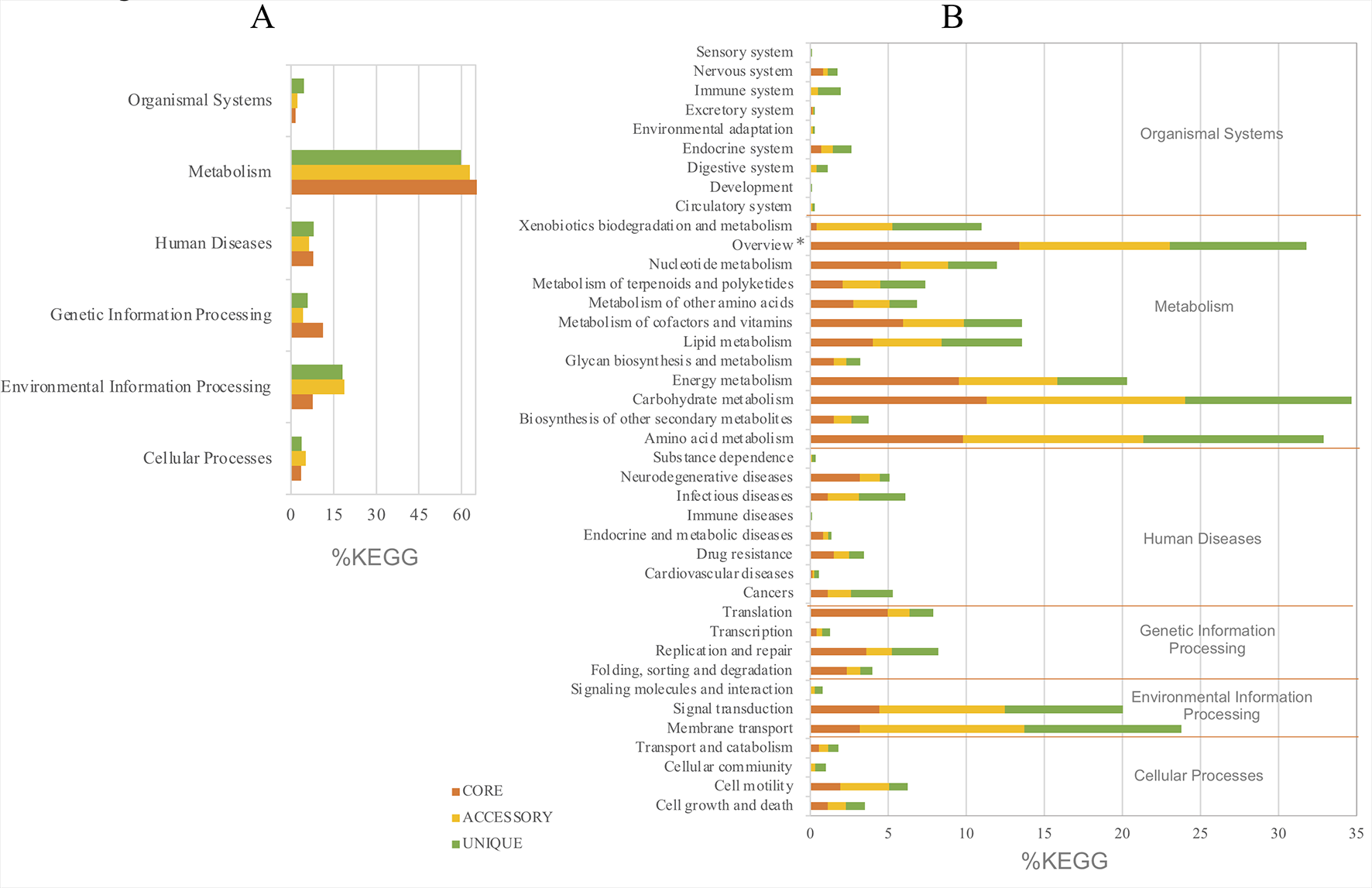

Supplement: Supplementary Figure 3 — Functional annotation and distribution of core, accessory, and unique genes of the PPFMs within KEGG main groups. (A) KEGG general category, and (B) more specific details of KEGG category. The gene count in each category is shown as a percentage of the total count in each group (core, accessory, and unique). ∗The overview category includes carbon metabolism (ko01200), oxocarboxylic acid metabolism (ko01210), fatty acid metabolism (ko01212), degradation of aromatic compounds (ko01220), and biosynthesis of amino acids (ko01230). [file Image_3.TIFF]

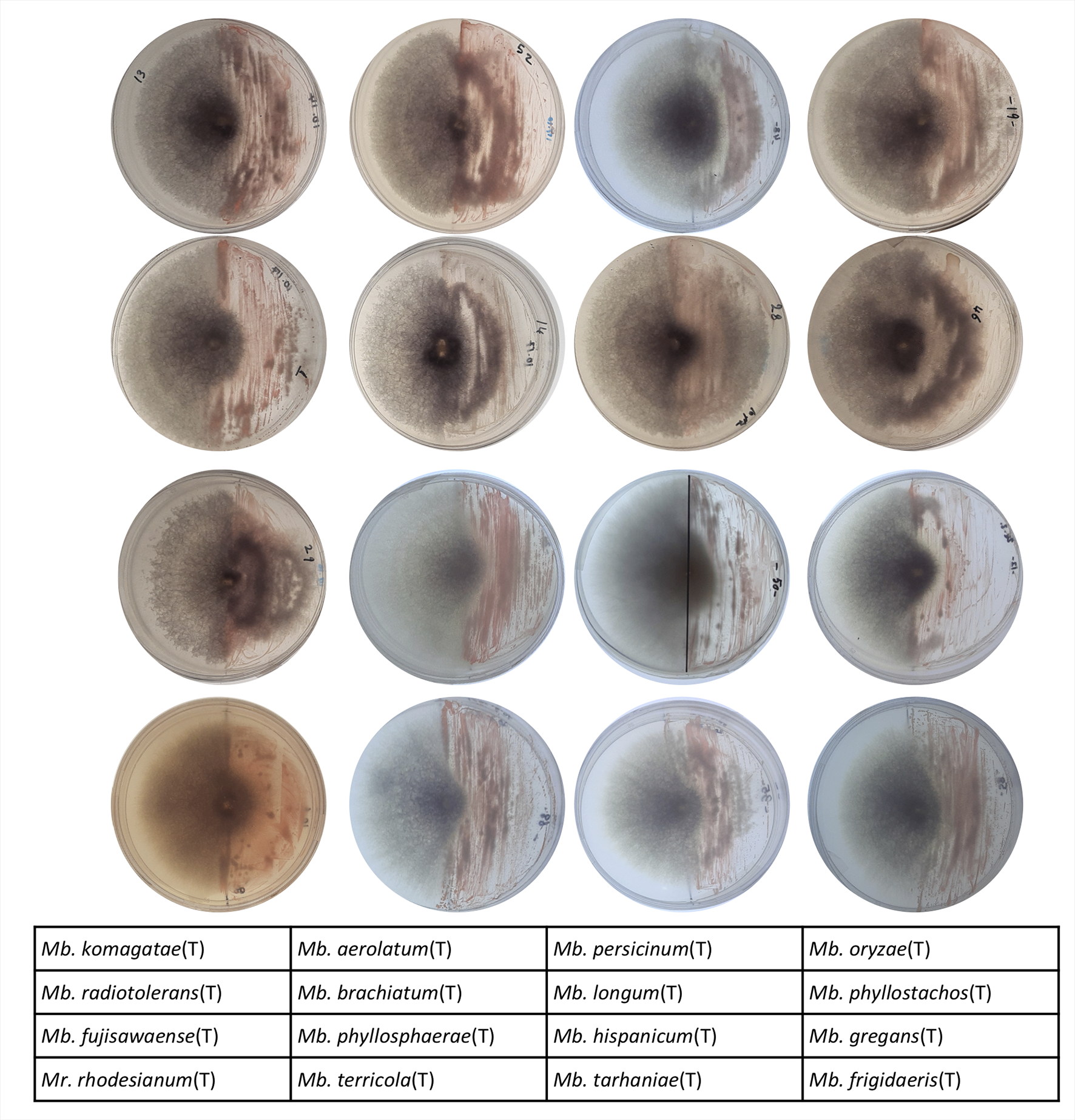

Supplement: Supplementary Figure 4 — Antifungal activity of PPFMs against Fusarium oxysporum. Among the tested strains, only those with strong inhibition activity are shown. Each PPFM was inoculated onto half of a PDA plate, and the fungus was allowed to grow from the center. The plates were incubated at 25°C for 7–10 days. [file Image_4.TIFF]

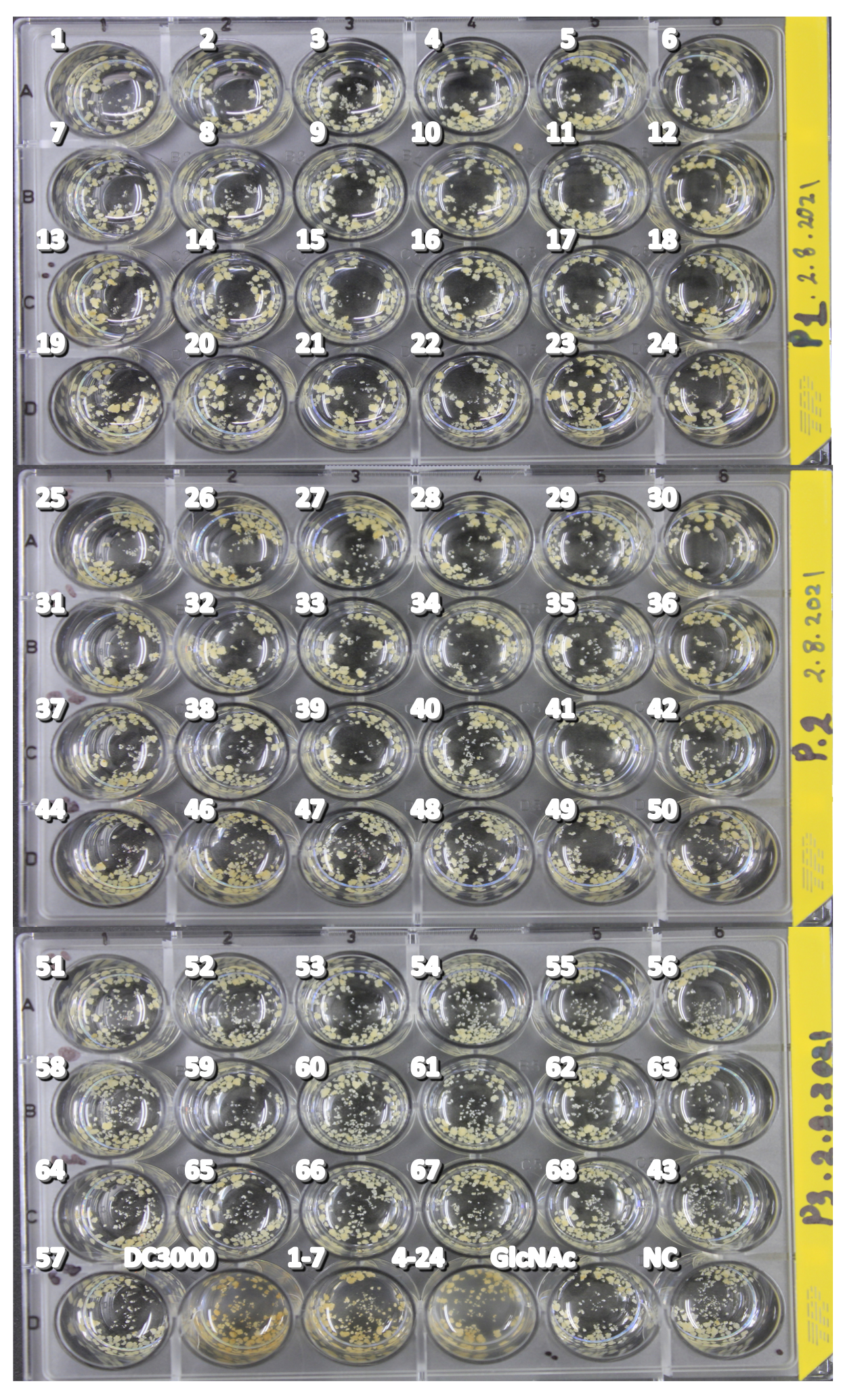

Supplement: Supplementary Figure 5 — Rice cell elicitation assay. The PPFMs were inoculated into rice cell suspension, then incubated for 24 h. The numbers represent the tested species listed in Supplementary Table 13. DC3000, Pseudomonas syringae DC3000; 1–7 and 4–24, Serratia marcescens subsp. marcescens isolates 1–7 and 4–24; GlcNAc, chitin oligomer (10 nM); and NC, negative control (saline solution). [file Image_5.TIFF]
